# Temperature variability and other climatic attributes linked to genomic features in the lichen-forming fungal genus *Umbilicaria*

**DOI:** 10.1186/s12915-025-02373-x

**Published:** 2025-10-02

**Authors:** Edgar L.Y. Wong, Anjuli Calchera, Jürgen Otte, Imke Schmitt

**Affiliations:** 1 Senckenberg Biodiversity and Climate Research Centre, Frankfurt Am Main, Germany; 2 Goethe University Frankfurt, Department of Biosciences, Institute of Ecology, Evolution and Diversity, Frankfurt, Germany

**Keywords:** Adaptation, Comparative genomics, Freezing, Phylogenomics, Methylation rate, Symbiosis

## Abstract

**Background:**

Many species of lichen-forming fungi exhibit large geographical ranges and broad thermal niches, making them excellent models for investigating the genomics of climate adaptation. In this study, we examined the impacts of climatic variables on genomic features in 11 *Umbilicaria* species. We compared PacBio genomes of individuals from the same species collected in different climate zones: alpine, cold temperate, or Mediterranean.

**Results:**

Our findings revealed several links between climatic and genomic features: (1) Selection pressure: in each climate zone, specific genes are under strong selection. (2) Genomic feature correlations: certain temperature variables (BIO2: mean diurnal range, BIO4: seasonality, BIO6: minimum in coldest month, BIO7: annual range) are correlated with GC content and the usage of the amino acids arginine and valine, suggesting these variables may drive convergent evolution of these genomic features. (3) Temperature variability: bioclimatic variables representing temperature variability, e.g. BIO2,4,7 are more influential in shaping genomic features than temperature means or extrema, with BIO6 also playing a significant role. (4) Epigenetic modifications: the rate of 5-methylcytosine (5mc) methylation within species is generally higher in samples from the colder habitat, suggesting that epigenetic modifications may contribute to climate adaptation.

**Conclusions:**

Overall, our study shows that genome evolution is partially shaped by climate and, particularly, temperature variability. This aligns with numerous ecological and climate modelling studies, which show that climate variability has a stronger impact on species behaviour and evolution than climate means and extrema. Further genomics studies are required to provide additional evidence on this topic.

**Supplementary Information:**

The online version contains supplementary material available at 10.1186/s12915-025-02373-x.

## Background

Climate is a key driver of the evolution and distribution of species. Numerous studies have shown that climate-induced changes in habitat availability and limited adaptive genomic potential can lead to range contractions and shifts, and even extinction [1–5]. Climate change can also contribute to the loss of fitness in populations [6] and changes in population genomic composition [7, 8]. Environmental changes can lead to rapid maladaptation, sometimes within a single year, as demonstrated in trout moved to captivity [9]. However, for species with wide distributions, local adaptation across extensive environmental gradients may provide pre-adaptive alleles. These alleles can potentially mitigate maladaptation through gene flow, thus enhancing species resilience to climate change [10]. Hence, it is crucial to understand the underlying mechanisms of climate adaptation in order to identify relevant traits and predict species responses.

Numerous genomic features have been linked to climate adaptation. Many studies categorise organisms based on climate proxies such as optimal or maximum growth temperature [11–14], overall temperature affinity (e.g. cryophile, mesophile: [15, 16], or habitat type of their sampling location [17]). Some of these studies found that specimens from the same biomes shared similar codon and amino acid usage [17], which has been proposed to have direct effects on the speed and accuracy of tRNA translation especially at low temperatures [18–20]. Heat-adapted organisms have been linked to higher proline and arginine content in proteins [21–23], higher GC content [12, 24], higher frequency of AGG (arginine) codons [15], and lower frequency of CGT (arginine) codons [15]. On the contrary, cold-adapted species showed increased catalytic efficiency in enzymes [25], higher alanine, glycine, glutamine, serine, and threonine content in proteins [24, 26], lower leucine content [26, 27], higher levels of GGA (glycine) and CGA (arginine) codons [20], and higher levels of cryoprotectants (e.g. anti-freeze proteins and trehalose) [28, 29]. However, there is no consensus on universal features that describe organisms with specific climate affinities, and results vary depending on the taxa. For example, GC content was found to have variable trends with optimal growth temperature, and between thermo- and non-thermophilic organisms depending on codon positions [13, 15]. In plants under stress, there is a tendency for increased GC content, stronger GC bias at the third codon position, and stronger codon usage bias in highly expressed genes [30]. While these studies provide a basis for studying climate adaptation, the parameters used often do not provide concrete details on the actual environmental characteristics experienced by organisms, nor take into account the effect of variability of climate.

Climate variability comprises numerous parameters that describe the range of climatic conditions an organism experiences over a given time period, such as temperature and precipitation. It has long been proposed that climate variability is more important than climate means and extrema in predicting extreme weather events (e.g. [31]). For instance, species with broader niche breadths (defined as the range of niches experienced/tolerated by an individual, population, or species) were found to have a higher ability to buffer the effects of climate change [32, 33]. Additionally, incorporating climate variability has been shown to improve climate modelling predictions [32, 34]. However, many species distribution models fail to account for climate variability [35–37], despite its implications for conservation, biological invasion, food security, and disease control [38–40]. Studying species adapted to highly variable climatic conditions, such as those with a large geographic range or broad niche breadth, would provide invaluable insights into the morphological, physiological, and genomic basis of environmental versatility and resilience. This is increasingly important given the higher frequencies of drastic weather events under accelerated climate change.

Several studies have dissected the relationship between niche breadths and fitness trade-offs [41–43], or between niche breadths and physiological performances [44–49]. However, only few genomic studies exist in this area: [50] found a correlation between genome size and temperature or salinity breadth in reef fishes; [51] found a correlation between carbon niche breadth and the number of annotated coding sequences or KEGG ortholog groups; [52] and [53] found gene expression differences between sites of different temperature breadths in crabs and salinity breadths in bacteria respectively.

In this study, we investigated the potential impact of climatic parameters, including climate variability, on genome evolution using 11 species of lichen-forming fungi in *Umbilicaria* (Umbilicariaceae). We identified genomic features that distinguish samples of the same species originating from different climate zones and pinpointed specific climatic variables linked to genomic differentiation. *Umbilicaria* Hoffm. is a genus of obligate lichenised fungi that forms rock-dwelling lichens with *Trebouxia* species (green algae) as photobionts [54]. All species in this study occur across at least two climate zones (alpine, cold temperate or Mediterranean).

Notably, some species of *Umbilicaria* exhibit exceptional cold tolerance with regard to gas exchange and photobiont activity. For example, *U. alpina* was found to be photosynthetically active at − 17 °C in Antarctica [55]; *U. pustulata* and *U. spodochroa *were capable of having unhindered carbon dioxide exchange under ice formation on thalli, and southern Norwegian individuals of the former had active photosynthesis during a whole frost day [56]. Previous studies on *U. phaea* and *U. pustulata* revealed genetic differentiation between populations from high and low elevations, corresponding to cold temperate and Mediterranean climate zones respectively [57, 58]. These high and low elevation populations exhibited differences in biosynthetic gene clusters, circadian-related genes, protein families, and genome-wide polymorphisms [59–63]. Besides, changes in photobiont entities and the composition of the lichen-associated microbiome (bacteria, viruses, other fungi) were evident among populations from different elevations [64, 65].

In lichens, mycobionts may pair with different photobiont taxa in different environments [58, 64, 66–68]. While some photobiont taxa are adapted to different environmental niches, it remains unclear how the same mycobiont species could tolerate a wide range of environmental conditions, and how their symbiotic interactions with photobionts enhance their environmental versatility. To address the former, we hypothesise that samples collected from different climate backgrounds would exhibit different, potentially convergent, genome characteristics. Specifically, we aim to include parameters that describe climate variability to test whether there is a correlation between niche variability (breadth) and genome evolution. We ask: (1) Which genomic features are associated with climate? and (2) Which bioclimatic variables influence genomic features and genome evolution?

## Results

### Features and phylogenomic relationships of the 27 highly complete genome assemblies

**Fig. 1:**
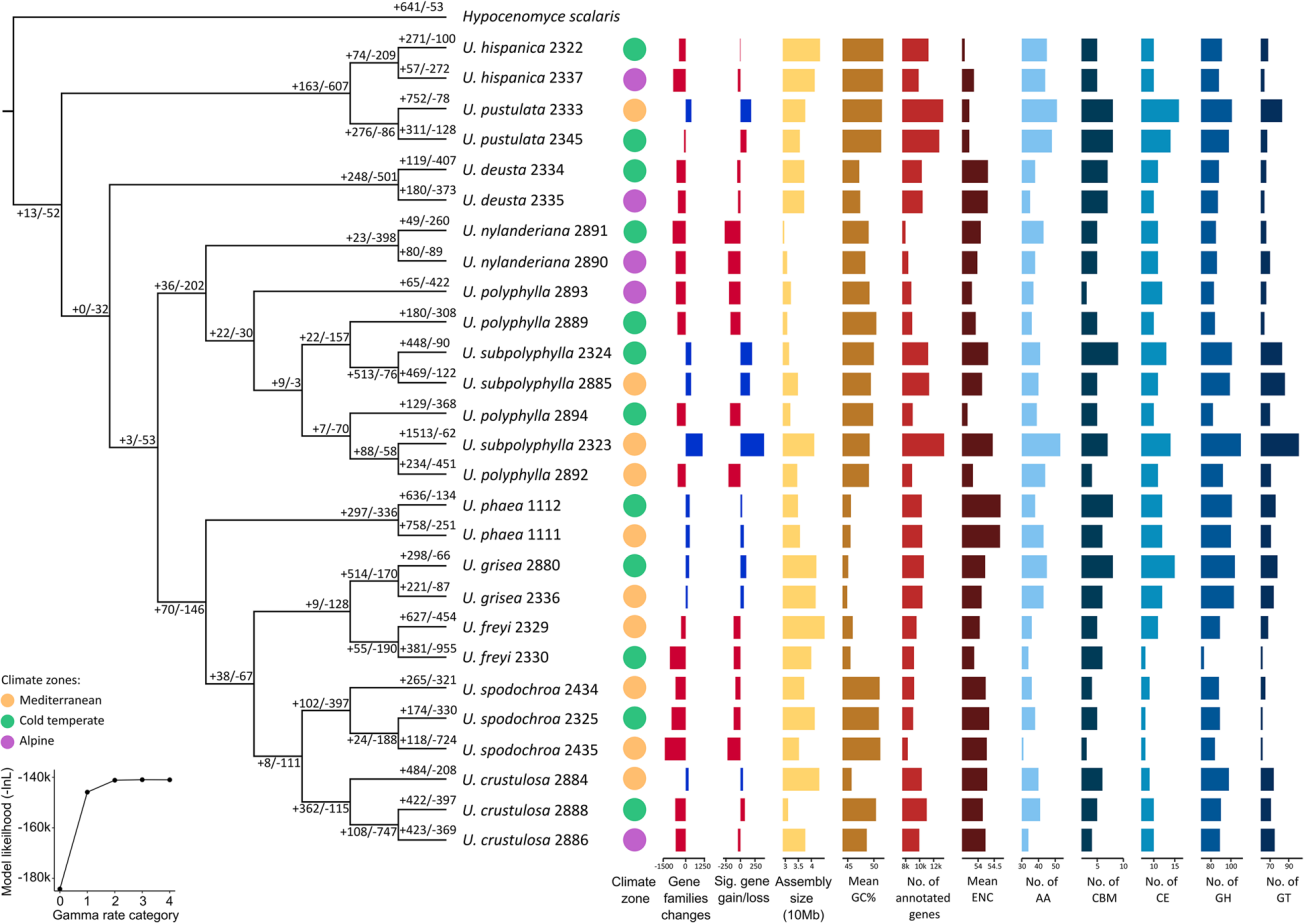
Phylogenomic relationship among samples (based on 15,306 orthogroups) and analyses on gene family changes along the phylogeny. Phylogenetic relationships were inferred from concatenated alignments of all orthologous groups detected by OrthoFinder. +/- numbers at each node on the phylogeny denotes the number of expanded and contracted gene families (significant and insignificant). See Additional File 2: Figure S1 for phylogram. Line graph on the bottom left shows the likelihood of each gamma rate category for CAFE5 analysis (gamma model *k *= 1 was used in subsequent analysis). On the right side of the phylogeny, various features of the genomes are shown: climate zone where the sample was collected, net gene family changes, net gene gain or loss from gene families with significant changes, assembly size, mean GC%, number of annotated genes, mean effective number of codons (ENC), number of annotated CAZymes in each enzyme class [auxiliary activities (AAs), carbohydrate-binding modules (CBMs), carbohydrate esterases (CEs), glycoside hydrolases (GHs), glycosyltransferases (GTs)]. No polysaccharide lyases (PTs) were annotated for any sample

All 27 newly assembled, re-assembled or re-filtered fungal genomes (Table 1, Additional File 1: Table S1) are highly complete, with 85–99.5% core fungal genes identified by BUSCO. A phylogeny based on 15,306 orthogroups revealed that each species is monophyletic, except for *U. polyphylla* and *U. subpolyphylla*, which together form a monophyletic clade intermixed with individuals from both species (Fig. 1a, Additional File 2: Figure S1). All newly or re-assembled genomes are gapless (no scaffolding was done) (Additional File 2: Figure S2). Genome sizes range from 29.6 to 45.3 Mb. GC contents range from 45 to 52%. The number of annotated genes ranges from 7965 to 12,839 (Additional File 1: Table S1). The detection of telomere ends in the assembled contigs (a typical feature of the end of chromosomes) reveals that many contigs in the genomes contain both telomere ends.

**Table 1.** Details of samples in this study. More details on assembly quality and RNA sequences can be found in Additional File 1: Table S1

**Species**	**Sample ID**	**Climate zone**	**Sampling country**	**Elevation (masl)**	**Assembly size (Mb)**	**No. scaffolds**	**No. annotated genes**	**Complete BUSCO%**	**Genome accession no.**
*U. crustulosa*	TBG2884	Mediterranean	France	553	43.1	44	9,349	99.3	JBNORA000000000
	TBG2888	Cold temperate	France	1250	31.1	51	10,136	85.0	JBNORC000000000
	TBG2886	Alpine	France	2450	37.7	35	9,243	88.1	JBNORB000000000
*U. deusta*	TBG2334	Cold temperate	France	1360	37.3	29	10,155	95.4	JBNORD000000000
	TBG2335	Alpine	France	2350	37.2	29	10,275	96.9	JBNORE000000000
*U. freyi*	TBG2329	Mediterranean	France	425	45.3	73	9,552	95.4	JBNORF000000000
	TBG2330	Cold temperate	France	1250	40.0	28	8,997	88.1	JBNORG000000000
*U. grisea*	TBG2336	Mediterranean	France	425	41.6	31	9,492	99.2	JBNORH000000000
	TBG2880	Cold temperate	France	80	41.8	52	9,510	99.5	JBNORI000000000
*U. hispanica*	TBG2322	Cold temperate	Spain	1504	43.3	71	11,262	98.7	JBNORJ000000000
	TBG2337	Alpine	Spain	2154	41.3	31	9,850	98.8	JBNORK000000000
*U. nylanderiana*	TBG2891	Cold temperate	France	1359	29.6	22	7,965	99.4	JBNORP000000000
	TBG2890	Alpine	France	2350	30.7	23	8,369	99.4	JBNORO000000000
*U. phaea*	TBG1111	Mediterranean	USA	631	35.6	40	9,236	97.6	JBNORQ000000000
	TBG1112	Cold temperate	USA	2036	34.9	26	8,995	99.5	JBNORR000000000
*U. polyphylla*	TBG2892	Mediterranean	France	330	34.6	82	8,915	99.5	JBNORT000000000
	TBG2894	Cold temperate	France	1250	31.9	34	8,998	99.5	JBNORV000000000
	TBG2889	Cold temperate	France	1688	30.7	67	8,932	99.4	JBNORS000000000
	TBG2893	Alpine	France	2350	32.1	26	8,803	99.3	JBNORU000000000
*U. pustulata*	TBG2333	Mediterranean	Spain	659	37.6	27	12,839	99.0	JBNORW000000000
	TBG2345	Cold temperate	Spain	1504	35.2	23	11,847	98.7	JBNORX000000000
*U. spodochroa*	TBG2434	Mediterranean	Spain	659	37.2	69	9,207	99.3	JBNORZ000000000
	TBG2435	Mediterranean	Spain	659	35.2	73	8,263	94.7	JBNOSA000000000
	TBG2325	Cold temperate	Spain	1504	41.3	268	9,061	99.1	JBNORY000000000
*U. subpolyphylla*	TBG2323	Mediterranean	France	330	41.1	87	12,485	99.5	JBNORL000000000
	TBG2885	Mediterranean	France	700	34.8	31	9,995	99.3	JBNORN000000000
	TBG2324	Cold temperate	France	1350	31.4	30	10,018	99.3	JBNORM000000000

#### Significantly expanded or contracted gene families

The orthogroups identified by *Orthofinder* were analysed by *CAFE 5* (Additional File 1: Table S2) with the gamma model *k* = 1. Although higher *k* values resulted in slightly higher likelihoods, they resulted in gene families with failures in the analyses (in which *k* = 1 resulted in none) and were hence neglected. Seven hundred three (4.59%) gene families (orthogroups) underwent significant expansion or contraction (hereafter CAFE families). No gene ontology (GO) terms were enriched in genes within CAFE families (Additional File 1: Table S3).

*U. polyphylla* and *U. subpolyphylla* were previously shown to be monophyletic species and closely related [54]. In our study, all *U. polyphylla* samples showed a net decrease in genes and gene families (there were more gene family contractions than expansions), whereas all *U. subpolyphylla* samples showed a net increase in both features, as well as higher effective number of codons (ENC) than *U. polyphylla* (Fig. 1a) (details in Additional File 2).

#### Genes under positive or negative selection in each climate zone

**Fig. 2:**
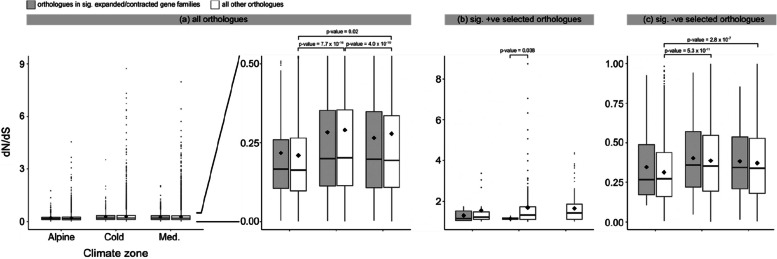
Test for selection among samples collected from each climate zone, compared between orthologues in gene families with significant expansion or contraction and all other orthologues. **a** dN/dS distribution in all orthologues identified by OrthoFinder (full figure on the left and zoom-in figure on the right for better resolution). **b** dN/dS distribution in all genes under significant positive selection (1 ≤ dN/dS < 10). **c** dN/dS distribution in all genes under significant negative selection (0 < dN/dS < 1)

Overall, orthologous genes showed significant differences in dN/dS between all pairs of climate zones, with dN/dS highest in the cold temperate zone, followed by the Mediterranean and alpine zone (see Fig. 2 for *p*-values). There is no difference in dN/dS for orthologous genes in CAFE family genes (Fig. 2a).

Orthologous genes under significant positive selection included 22 genes in the alpine, 145 in the cold temperate, and 102 in the Mediterranean zone (Additional File 1: Table S4). Among these genes, only a few had an annotated gene products that were not a hypothetical proteins: two for the alpine zone, five for the cold temperate zone, and none for the Mediterranean zone (Fig. 2b, Additional File 1: Table S4). No CAFE family genes were under significant positive selection in the Mediterranean zone (Fig. 2b). In the cold temperate zone, dN/dS was significantly lower in CAFE family genes than in other genes (Fig. 2b).

In contrast, orthologous genes under significant negative selection included 716 genes in the alpine, 1763 genes in the cold temperate, and 1621 genes in the Mediterranean zone (Fig. 2c, Additional File 1: Table S4). Among these, CAFE family genes did not show differences between each pair of climate zones, whereas in other genes the alpine zone samples had significantly lower dN/dS than both the cold temperate and Mediterranean zone samples (Fig. 2c).

### Genes associated with secondary metabolism

All data described in this section can be referred to in Additional File 1: Table S5-S10.

Genes associated with secondary metabolism were found to be highly enriched in genes under positive or negative selection in certain climate zones. These include non-reducing polyketide synthase (PKS8-1), which is under negative selection in the alpine zone for two species (udeu2335_005949, uhis2337_003682) (Additional File 1: Table S3, S8). A sterol O-acyltransferase 2 (sterol-ester synthase 2) was under negative selection in both the cold temperate zone (ucru2888_001396, udeu2334_002352, ufre2330_001743, ugri2880_001145, uhis2322_005074, usub2324_003306, unyl2891_002958, upha1112_001938, upol2894_001884, upus2345_008950) (Additional File 1: Table S9) and the Mediterranean zone (ucru2884_003544, usub2323_003248, usub2885_002428) (Additional File 1: Table S10). Additionally, genes under negative selection in the Mediterranean zone included a mevalonate kinase (ucru2884_000326, ufre2329_004626, ugri2336_002050, usub2885_001993, upha1111_000341, upol2892_003566, upus2333_001215, uspo2434_001485, uspo2435_000318) and a lanosterol 14-alpha-demethylase (usub2885_001855). The gene family trees (inferred by OrthoFinder) for these four orthologous groups can be found in Additional File 2: Figure S3.

#### Climate-related GO enrichment in genes under selection

**Fig. 3:**
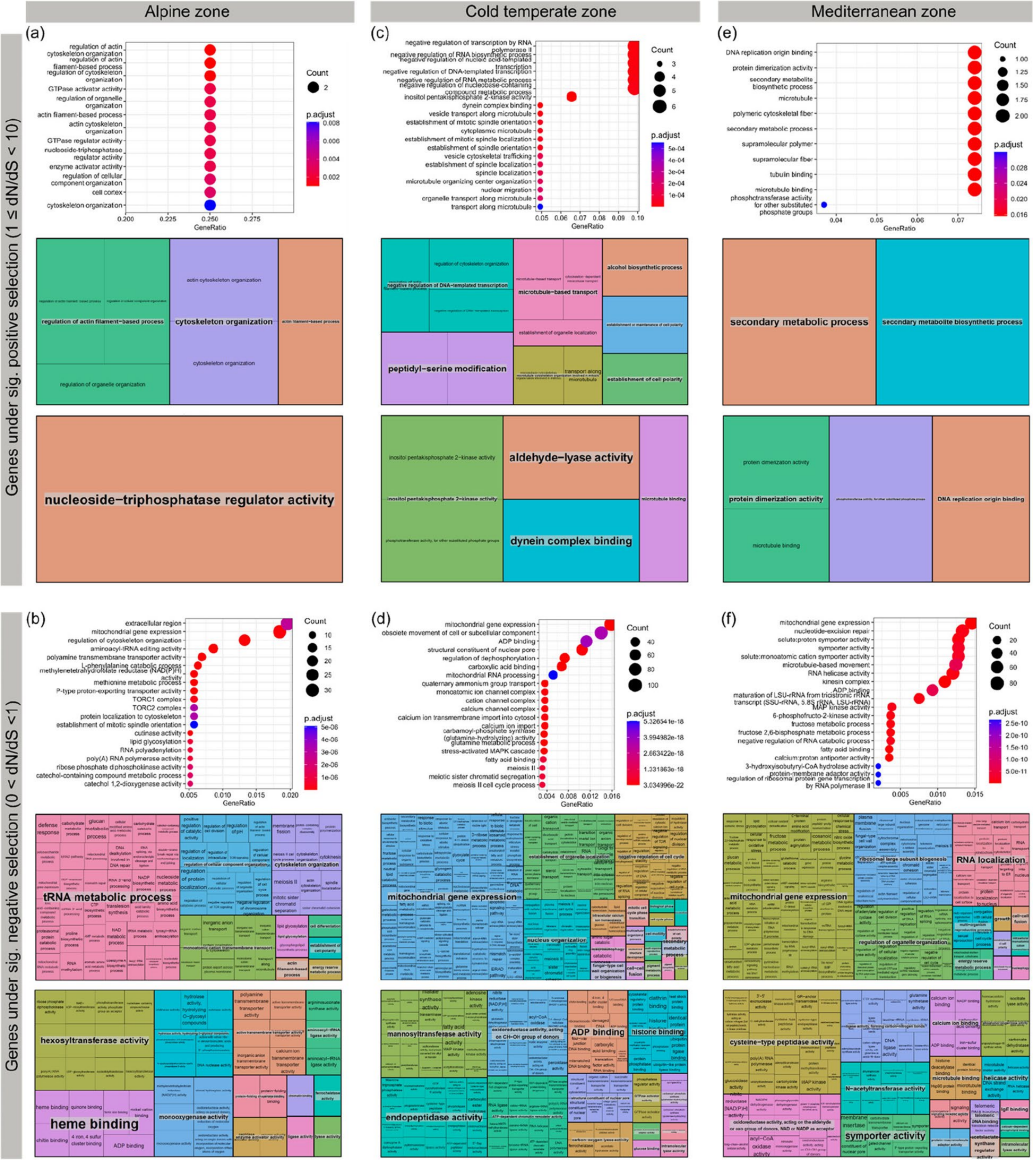
Enriched GO terms in genes with significant positive or negative selection among samples in each climate zone. For each panel, the top figure shows the top enriched GO terms, with circle size denoting the number of genes involved. The middle and bottom figure show the summary of enriched GO terms for biological functions (middle) and molecular functions (bottom) respectively. Each colour denotes a GO category with the category name in grey background, and each box represents different GO terms under each category

All data described in this section can be referred to in Figs. 1b–d and 3 and Additional File 1: Table S4-S12.

#### Genes under significant positive selection

In the alpine zone, actin-based processes and cytoskeleton organisation were the only two groups of enriched GO terms (Fig. 3a). In the cold temperate zone, the contributing groups were negative regulation of DNA-templated transcription, microtubule processes, alcohol biosynthetic processes, cell polarity, and peptidyl serine modification (Fig. 3c). In the Mediterranean zone, all enriched GO terms are related to secondary metabolites for biological processes and supramolecules for cellular components (Fig. 3e).

#### Genes under significant negative selection

In the alpine zone, biological processes such as tRNA metabolic processes contribute to almost half of the enriched GO terms, with cellular component organisation, cytoskeleton organisation, actin filament-based process, and monoatomic cation transmembrane transport also contributing to substantial amount (Fig. 3b). In terms of molecular functions, activities regarding hexosyltransferase, monooxygenase, active transmembrane transporter, hydrolase, ligase, lyase; as well as heme binding were the major contributors (Fig. 3b). In the cold temperate zone, biological processes of mitochondrial gene expression are the largest group of enriched GO terms, followed by organelle localisation, negative regulation of cell cycle, nucleus organisation and other smaller contributions (Fig. 3d). Regarding molecular functions, major contributors include binding of ADP and histone, as well as activities of endopeptidase, mannosyltransferase, chromatin remodeler and oxidoreductase (Fig. 3d). In the Mediterranean zone, biological processes of mitochondrial gene expression are also the largest group of enriched GO terms, followed by ribosomal large subunit biogenesis, RNA localisation, organelle organisation and other smaller contributions (Fig. 3f). For molecular functions, binding of calcium ion and microtubule, as well as activities of cysteine-type peptidase, symporter, helicase, oxidoreductase, ligase, and N-acetyltransferase, were the major contributors (Fig. 3f).

#### Enriched GO terms of genes under selection that are shared by more than one climate zone

For genes under positive selection, the alpine zone does not share any enriched GO terms under positive selection with the Mediterranean zone. Between the alpine and cold temperate zone, all three shared enriched GO terms are related to the cytoskeleton. Shared GO terms between the cold temperate and Mediterranean zones include those related to supramolecules and microtubules. For genes under negative selection, many enriched GO terms are shared among all three climate zones, such as those related to calcium ion, negative regulation of cell cycle, cytoskeleton and arginine biosynthetic process. Eleven enriched GO terms are shared between the alpine and cold temperate zones, including those involving various enzymes (DNA exonuclease, GTPase, ferrochelatase, glucosyltransferase, tRNA ligase), DNA damage activity and chitin binding. For enriched GO terms that are shared between the cold temperate and Mediterranean zone, examples include those related to exoskeleton, various metabolic processes (e.g. fructose, chitin and glycine), oxidative or osmotic stress responses. No GO terms are shared by just the alpine and Mediterranean zone for both positively or negatively selected genes.

### Relationship between climatic factors and genomic features

Comparing amino acid compositions between sample pairs from different climate zones within the same species, all pairs showed significant differences in alanine, cysteine, glycine, and threonine compositions (Additional File 2: Figure S4-S5).

**Fig. 4:**
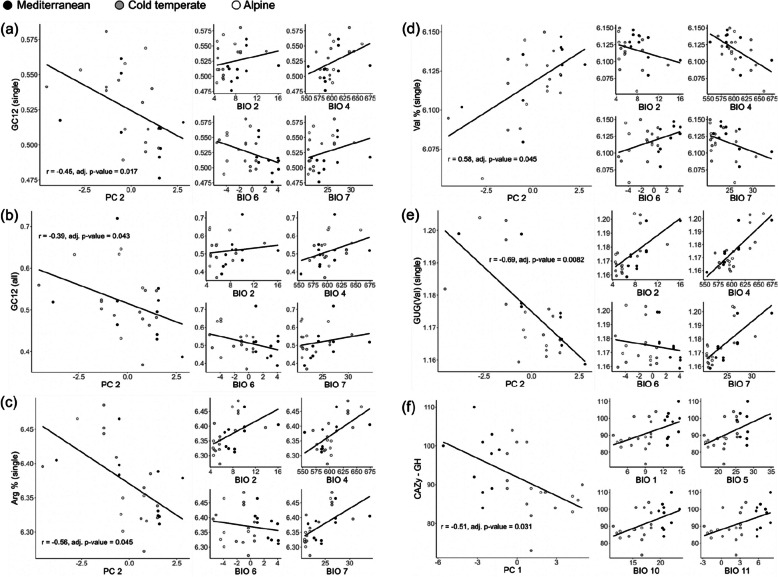
Correlations between bioclimatic variables (grouped by principal components) and genomic features. Genomic features correlated with either PC2 (**a–e**), or PC1 (**f**). For each panel, the left figure shows the correlation between genomic feature and PC. The four smaller figures on the right show the relationships between the same genomic feature and bioclimatic variables that contributed to > 10% of that PC (see Additional File 2: Figure S8 and Additional File 1: Table S14-S15 for details). All genomic features were estimated from single-copy orthologues except (**b**) GC12 and (**f**) CAZymes (GH), which were estimated from all orthologues

In the principal component analysis (PCA), almost all significant correlations were associated with principal component (PC) 2 which harbours bioclimatic variables that describe temperature variability (BIO2: mean diurnal range, BIO4: temperature seasonality and BIO7: temperature annual range) and freezing probability (BIO6: minimum temperature of coldest month), but not PC1 which harbours variables that describe temperature means and extrema (BIO1: annual mean temperature, BIO5: maximum temperature of warmest month, BIO10: mean temperature of warmest quarter and BIO11: mean temperature of coldest quarter) (see Additional File 2: Figure S6 and Additional File 1: Table S13-S14 for loading values and contribution of bioclimatic variables to each PC). Correlation analyses using PC coordinates showed no correlation between genomic features with PC3 (major contributors being BIO8: mean temperature of wettest quarter and BIO9: mean temperature of driest quarter) (Additional File 1: Table S15). Five genomic features showed significant correlation with PC2, namely GC12 (in both single-copy orthologues and all other orthologues), arginine (in single-copy orthologues), valine (in single-copy orthologues) and GUG (valine) (in single-copy orthologues) (Fig. 4a–e; see Additional File 1: Table S15 for *r* and *p*-values). GC12, arginine, and GUG increased with BIO2, BIO4 and BIO7, but decreased with increasing BIO6 (Fig. 4a–c,e). Valine showed the opposite trend: it decreased with increasing BIO2, BIO4 and BIO7, while it increased with BIO6 (Fig. 4d). Only the size of the GH family (CAZymes) correlates with PC1. It increases with BIO1, BIO5, BIO10 and BIO11 (Fig. 4f). Loading values for each bioclimatic variable and contributions of bioclimatic variables to each PC can be found in Additional File 1: Table S13-14.

**Fig. 5:**
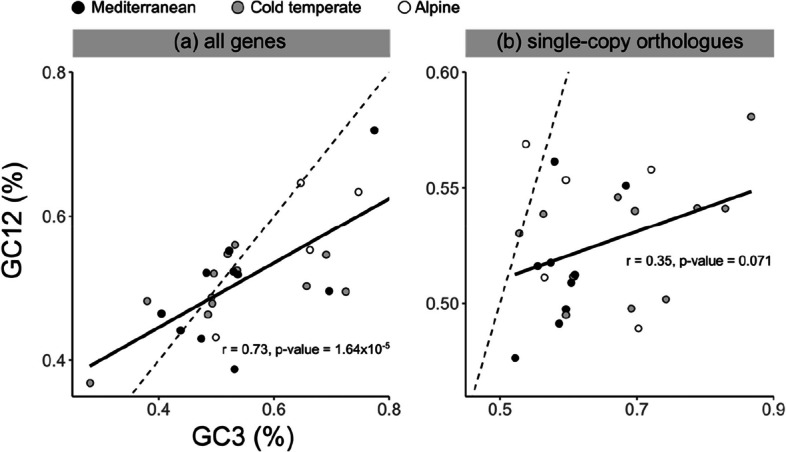
Neutrality plot showing the correlation (or lack thereof) between GC content in the first two codon positions (GC12) and the third codon position (GC3) in (**a**) all genes and (**b**) single-copy orthologues only. The black line denotes the relationship between GC12 and GC3. The dotted line denotes the hypothetical 1:1 relationship between the two

GC content (GC12 and GC3) (Additional File 2: Figure S7), ENC (Additional File 2: Figure S8) and CAZymes in each class (Additional File 2: Figure S9) did not differ among climate zones. However, neutrality plots revealed that GC12 has a significant positive correlation with GC3 among all annotated genes (*r* = 0.73, *p*-value = 1.64 × 10^−5^), but not among single-copy orthologues (Fig. 5). This suggests that natural selection has a stronger effect than mutation bias in single-copy orthologues, while the opposite is true for other genes. Correspondence analysis of relative synonymous codon usage did not show any clustering specific to climate zone (Additional File 2: Figure S10). However, *U. deusta* samples were separated from the rest of the samples in both gene categories; *U. pustulata* and *U. hispanica* were separated from the rest of samples among single-copy orthologues (Additional File 2: Figure S10). Among all annotated genes, *U. polyphylla* and *U. subpolyphylla* samples were distantly apart (Additional File 2: Figure S10). Overall, some bioclimatic variable values are significantly different between the Mediterranean zone and alpine or cold temperate zone (Additional File 2: Figure S11). There is no significant difference between the alpine and cold temperate zone (Additional File 2: Figure S11).

### Methylation level differentiation within species

We compared 5-methylcytosine (5mC) methylation levels (of 1 Mb windows) between individuals of the same species that were collected from different climate zones. In each of the seven analysed species, methylation levels were higher in the sample from the colder climate zone overall, but this was significant only in *U. crustolosa*, *U. deusta*, *U. subpolyphylla*, and *U. pustulata* (Additional File 2: Figure S12a, d, f). However, within the same species, methylation level was not always higher for all genomic windows in the individual from the colder climate zone. The methylation level is higher in the sample from the colder zone in all windows in *U. pustulata* only (Supp Fig. 12f). Besides, methylation levels vary greatly among windows and species (Supp Fig. 12). For instance, *U. pustulata* (cold temperate) has a methylation rate of over 0.012%, whereas *U. crustulosa* (alpine) had the highest methylation level at around 4.5 × 10^−4^% (Additional File 2: Figure S12a, g).

## Discussion

In this study, we identified numerous climate-related gene and functional targets in lichen-forming fungi. Notably, we also found more correlations between genomic features and temperature variability than between genomic features and temperature means or extrema. As widespread mutualisms that grow in all habitats from the Antarctic to the tropics, lichens are extraordinary symbioses that can be used to address both ecological and evolutionary questions regarding environmental adaptation, symbiosis, and how symbiotic interactions confer enhanced environmental versality. Future studies should aim at further dissecting patterns identified here, including those related to methylation.

### Potential effects of lichen holobiont composition and phylogenetic signal in observed patterns

Lichens are a symbiosis of mycobiont, photobiont, and associated microbiome that includes organisms such as other fungi, bacteria, and viruses. It is possible that the observed patterns in relation to climate reflect shifts in the composition of the holobiont, rather than direct responses to climatic conditions. Previous research has shown that climate zone has a role in shaping bacterial communities and photobiont associations within lichens [57, 64]. Studies examining changes in photobiont and microbiome compositions also found that the abundance of certain microorganisms (such as viruses) correlated with elevation [61, 64], which often serves as a proxy for certain climatic variables such as temperature. However, in the present study, genomic features of mycobionts (such as GC content and ENC) did not exhibit significant differences among zones (Additional File 2: Figure S7-S8). It is also noted that some photobiont taxa are climate generalists [69], occurring in multiple climate zones and associating with multiple mycobiont species. While we acknowledge that climate does influence holobiont compositions, these compositional changes appear to be linked to broad climate zone assignments, rather than correlate with changes in climatic conditions. Therefore, we conclude that the effects of lichen holobiont composition on the correlation between mycobiont genomic features and bioclimatic variables is likely minimal.

While the sample size in this study does not warrant reliable tests for phylogenetic signals in the correlations observed (see Additional File 2), the effects of phylogenetic relationships cannot be completely ruled out [70]. However, we deliberately selected samples of the same species from different climate zones, which come with different associated bioclimatic variable values and likely do not associate phylogenetically among samples. Hence, we conclude that the potential phylogenetic signals in the correlations observed would be minimal. In genomic features that do not correlate with bioclimatic PCs, phylogenetic signals in genomic features or other factors could explain the lack of correlation.

### Secondary metabolism

Lichens are known to produce many secondary metabolites, and GO terms associated with secondary metabolism are enriched in negatively selected genes from all climate zones (Fig. 3). The fact that purifying selection, i.e. the removal of detrimental mutations from populations, prevails in biosynthetic genes of lichen mycobionts suggests that these genes are crucial to the lichen lifestyle and non-synonymous mutations in these genes could lead to reduced fitness.

A non-reducing polyketide synthase (PKS8-1), which is exclusively annotated for samples from the alpine zone, is part of the biosynthetic pathway of dibenzodioxocinones, a derivative of anthraquinones [71, 72]. Anthraquinones are produced by *Umbilicaria* species, and have been linked to cold tolerance in thermophilic fungi [73] and plants [74]. Thus, it is possible that this gene underlies cold responses in alpine *Umbilicaria* individuals.

The other three predicted gene products: sterol O-acyltransferase 2, mevalonate kinase and lanosterol 14-alpha-demethylase are all involved in different modules of the ergosterol biosynthetic pathway [75–77]. Interestingly, farnesyl pyrophosphate synthetase, the protein product most annotated for genes with strong codon bias (ENC ≤ 35) (20 out of 27 samples) in our analysis (Additional File 2: Figure S13b), is also involved in this pathway [78–80]. Ergosterol (provitamin D_2_) is one of the major components of plasma membranes of both lichen mycobionts [81] and photobionts [82, 83]. It is a precursor to ergocalciferol (vitamin D_2_), and the conversion from ergosterol to ergocalciferol is triggered by ultraviolet (UV). Ergocalciferol content was shown to correlate with UV-B radiation in the lichen *Cladonia arbuscula* [84] and button mushrooms [85]. In other fungi, ergosterol has been shown to be involved in stress response [86, 87]. In particular, ergosterol facilitates membrane preservation under physical and chemical stress caused by drying and wetting [88]. Modifications to ergosterol in higher fungi have been shown to be tRNA dependent [89], and in the present study tRNA metabolism is the major GO category in negatively selected genes in the alpine zone. Building on these findings, we hypothesise that GO terms associated with ergosterol are enriched in *Umbilicaria* due to ergosterol’s role in responding to UV and potentially other environmental stressors. However, the precise functions of ergosterol, and any structural or functional variations in ergosterol produced by individuals from different climate zones, potentially arising from non-synonymous mutations, remain to be elucidated by future research.

### Genes and functions important to survival in different climate zones

Positive selection enables the spread of advantageous variants, while negative selection purges deleterious mutations, and maintains stability and functionality of important proteins. In our study, the former contributes to genomic differences between and within species and climate zones, whereas the latter likely affects genes universally important to all samples regardless of climate zones, such as genes related to symbiotic interactions and secondary metabolism.

Few genes are under significant positive selection (Additional File 1: Table S4). The mean dN/dS is negative for all three climate zones, both for CAFE family genes and for all other orthologues (Fig. 2a). Additionally, genes in the alpine zone experienced the strongest purifying selection (lowest mean dN/dS for all orthologues and significantly negatively selected orthologues; Fig. 2a, c). These findings suggest that genes of specimens from alpine niches are more conserved and that non-synonymous amino acid changes might incur higher fitness costs in these individuals. The absence of significant positive selection in CAFE genes in the Mediterranean zone also suggests these genes are important for adaptation to the colder climate.

Examples of supramolecular structures in fungi include complex molecular assemblies and architectures in cell walls [90], or respiratory chains [91]. In the present study, GO terms related to supramolecular structures were enriched in the Mediterranean zone, which leads us to speculate that these structures could play a role in heat and drought tolerance, or water retention capabilities of lichenised fungi. Supramolecular structures involving cell-wall associated melanin have been shown to potentially affect photoprotection and thermoregulation in fungi ([92, 93]. Under increasing salinity, filamentous fungi such as *Aspergillus sydowii* performed structural adjustments, leading to thicker and more rigid cell walls with limited water permeability [94]. Hence, the high number of GO terms related to supramolecules in *Umbilicaria* specimens collected from the Mediterranean zone might indicate the need for the modification of these molecules for survival under extended periods of drought.

### Important genes and functions in different climate zones

Enriched GO terms (Fig. 3, Additional File 1: Table S5-S12) related to environmental stress, particularly temperature stress, are present in all climate zones in different aspects of cell functions. These include GO terms related to cytoskeleton (including actin, actin filaments, actin depolymerisation proteins), calcium ion and chitin in all zones; microtubules and mitochondrial gene expressions in both the cold temperate zone and Mediterranean zone; and alcohol biosynthetic processes in the cold temperate zone. Genes with strong codon bias also show a high percentage of annotations related to cytoskeleton. Actin depolymerisation in plants [95, 96] and downregulated actin-related genes in fungi [97] have been linked to cold tolerance. Actin depolymerisation is a pre-requisite for cytoskeleton reorganisation [95, 98], which has a key role in the signalling pathways under cold stress, potentially through linking calcium influx and cell membrane rigidification to increase survival [95, 99, 100]. Like actin, microtubules are major components of the cytoskeleton and have been shown to be involved in stress sensing and response in plants and fungi [101–107]. In addition, sugar alcohol content was shown to change due to stress in fungi [108]. Chitin is an integral component of fungal cell walls, and it has been linked to cold tolerance [109] and temperature stress in fungi [110, 111]. In plants, a chitinase gene was found to be highly homologous to a gene encoding an antifreeze protein [112]. Mitochondrial gene expressions contribute to around one-third of the enriched GO terms in negatively selected genes in the cold temperate and Mediterranean zones. Although not extensively explored in lichen mycobionts, mitochondrial genes, including the divergence of their associated proteins, have been demonstrated to play a role in tolerance to both heat and cold in yeasts [109, 113, 114]. This finding could explain why these genes are under purifying selection in our study.

### Insights into adaptation to high environmental heterogeneity

We found overwhelming evidence from gene family changes, genes under selection and strong codon bias that indicate various aspects of temperature, especially temperature breadth (variability) and freezing probability, are strong selective forces driving genome evolution of the lichen-forming fungal genus *Umbilicaria*. We also showed intra-specific, climate-correlated differences in genomic features and methylation patterns. We found a generally higher methylation rate in samples from colder climate zones, which is in line with a previous study that showed higher 5mC methylation in fish adapted to polar temperatures compared to those adapted to tropical or temperate temperatures [115]. It is widely accepted that epigenetic processes are crucial for responses to climate variations, especially in organisms with long generation times whose rate of adaptation via genetic modifications is low [116–118]. However, both increase and decrease in methylation have been reported for both heat and cold stress for different genes [116, 119]. This could explain the variation in methylation rate within genomes and among species in this study. Our findings indicate that high genomic plasticity (variability) and/or few evolutionary constraints could be key for these widespread species to cope with high environmental heterogeneity. We established here the correlation between various genomic features (such as GC12, proportion of arginine, and valine) and bioclimatic variables, but the functional implications of these links require future research that tests the effect of climatic variables on specific genes and proteins.

Temperature niche breadth and minimum temperature appear to have opposite effects on genomic features, as we saw increase in genomic features (such as GC12 content) corresponds to increase in temperature variability but decrease in minimum temperature, and vice versa. These opposite patterns could be seen as opposing selective forces, just as the Climate Variability Hypothesis [120] and Climate Extremes Hypothesis [121] are often considered as mutually exclusive. The former predicts that organisms with a broader niche breadth would also be able to tolerate a wider range of climatic conditions (hence have a larger range), whereas the latter predicts that organisms that can tolerate higher climatic extremes would have a wider distribution. However, like [42], we believe these hypotheses should be complementary, as they focus on different aspects of thermal biology. The cumulative effect of different parameters is understudied and requires more attention.

Climate variability and climate extremes have both been researched extensively in ecological studies. However, in evolutionary studies especially those involving omics, climate variability is rarely considered while climate extremes are often used as proxies for tolerance or adaptation potential (see Introduction for examples). Although climate extremes inherently contribute to the degree of climate variability, focusing on extreme thresholds does not provide information on species responses to fluctuating climatic conditions. Only a handful of studies have looked at genomic or transcriptomic differences between samples from sites with different climatic niche breadths (in crabs: [52]; fish [50]; bacteria [53]; yeasts: [51]). Our finding that temperature breadth correlates with genomic features adds to this growing body of evidence. It also resonates numerous other studies that show environmental variability underlies differences in physiological performance and even heritability of tolerance (e.g. [44–49]). It also supports the notion that environmental variability is more important than means or extrema in predicting extreme weather events [31]. Besides, populations with broader niche breadths are likely more buffered from climate change [32, 33]. For instance, ‘stress memory’ could help individuals adapt to climate change better by enabling faster and stronger responses to previously experienced conditions [122, 123]. To demonstrate, it was found that cold stress induced higher methylation rates only in psychrotolerant, but not psychrophilic species of *Naganishia* fungi [124]. Regarding climate modelling, including climate variability was found to increase uncertainty for crop yields and others by 38% on average [34], whereas including genomic information in ecological niche modelling improved climate change predictions [32]. With many geographical regions on earth expected to experience a more variable climate in the future (e.g. precipitation variability: [125]), an increase in temperature variability poses a much greater risk to species than rising mean temperatures, especially for temperate species [38]. Changes in climate variability will also have social-ecological consequences, including impacts on food security [39] and human health, with the latter driven by the behaviour of disease vectors and their parasites [40]. Hence, we advocate for greater attention to the use of environmental variability parameters in omics studies to enhance our understanding of species’ tolerance and responses to environmental fluctuations.

## Conclusions

In this study, we assembled and analysed 27 high-quality genomes of lichen-forming fungi from 11 *Umbilicaria* species, sampled from multiple climate zones. We observed significant changes in gene families related to secondary metabolites across these samples. In each climate zone (alpine, cold temperate or Mediterranean), we identified gene functions that are unique to each zone or shared by several zones. The most striking finding of this study is that among the climatic variables that correlated with genomic features such as GC content and the usage of arginine and valine, most describe temperature breadth but not temperature means or extrema. This is consistent with a wealth of ecological and climate modelling studies, which showed that climate variability is a more important parameter than climate means or extrema. In line with previous studies, presence or absence of freezing has proven to be a key driver in genetic differentiation in *Umbilicaria* at both population and genus levels. Our findings suggest that climate variability is a prominent driver of genome evolution. Genotypes and phenotypes which are able to tolerate higher climate variability might be of higher conservational value than those that can only survive certain environmental thresholds. While climatic adaptation to fixed thresholds is well documented under experimental conditions, we believe the use of parameters related to environmental niche breadths (climate variability) would greatly improve our understanding of species response to accelerated climate change in the Anthropocene.

## Methods

All analyses were carried out using default setting unless specified below. Differences between groups in all analyses were tested using the parametric test analysis of variance (ANOVA) or non-parametric Kruskal–Wallis test depending on the normality of data. The parametric Tukey’s test and non-parametric Wilcoxon signed-rank test was used to further test pairwise differences if the previous tests showed significant differences (*p*-value < 0.5).

### Sampling for whole genome sequencing

Eleven *Umbilicaria* species were sampled in this study. We selected these species as they occur in different climate zones and could be sampled in close proximity on the same elevational gradient (except *U. grisea*) (Table 1). For each species, samples were collected from at least two climate zones in their range. Three climate zones were defined for the European and Californian sampling sites in this study based on characteristic vascular plant vegetation. The Mediterranean zone is characterised by the presence of helm oak (*Quercus ilex*) or cork oak (*Q. suber*) in Europe, and coast live oak (*Q. agrifolia*) in California. This zone extends from sea level to ~ 900 m at the European sites and ~ 1000 m at the Californian sites. The cold temperate zone is characterised by the presence of European beech (*Fagus sylvatica*) and black pine (*Pinus nigra*) at the European sites, and giant sequoias (*Sequoiadendron giganteum*) at the Californian sites. This zone ranges from ~ 900–1800 m at the European sites and ~ 1200–2400 m at the Californian sites. The alpine zone is located above the tree line, which is generally above 1900 m at the European sites. There is no alpine zone at our sampling locations in California.

We analysed 27 genomes, 15 of which have been reported before [126] (Table 1, Additional File 1: Table S1), but were either re-filtered or re-assembled using updated versions of programmes for genome assembly and contamination filtering. Briefly, contamination filtering included mapping against a reference genome, use of BlobTools [127] and FCS-GX [128] (see Additional File 2 for details of DNA and RNA extractions, genome assemblies and annotations) (Table 1, Additional File 1: Table S1).

### Gene family expansion and contraction

To investigate gene families that had undergone significant expansion or contraction among the samples we study, we used *CAFE 5* v5.1.0 [129], which uses a birth and death process to model gene gain and loss across a phylogeny. Prior to running *CAFE 5*, we used *OrthoFinder* v2.5.5 [130] with the multiple sequence alignments option (*-M* msa) to infer orthogroups and orthologous genes. *OrthoFinder* also infers a species tree using the STAG algorithm [131] on the concatenated sequence alignments. To aid in phylogenomic inference, we included annotations of *Hypocenomyce scalaris* (NCBI accession: ASM2281431v1), which is in the same order (Umbilicariales) as *Umbilicaria*, as an outgroup. After obtaining gene counts of all 15,306 orthogroups, three orthogroups with more than 100 gene copies were removed as recommended by the developers of *CAFE 5*. The species tree inferred by OrthoFinder was then converted to an ultrametric tree with branch lengths proportional to time using the *make_ultrametric.py* script in OrthoFinder and the root age set to 190 million years ago. The root age is based on the average of crown ages of Umbilicariales (which includes all *Umbilicaria* species and the outgroup *H. scalaris*) under four scenarios inferred by [132]. The resulting ultrametric tree and gene counts were analysed in *CAFE 5* using the base model and gamma model using model categories (*k*) of 1 to 4. The best fitting model was chosen by plotting likelihood values against model categories and selecting the model at which the likelihood curve starts to plateau. Using results from the selected model category, orthogroups with significant changes were analysed further.

### Selection analysis using dN/dS

To identify genes under positive and negative selection in each climate zone, we employed the dN/dS statistic, which is the ratio of non-synonymous to synonymous ratios. The *compare* command of the *funannotate* pipeline v1.8.15 was used to calculate dN/dS ratios in orthologous genes, with the flag *–run_dnds* set to full. The command was run for samples grouped by their respective climate zone of collection. Genes with dN/dS > 10 were discarded. Data was divided into two groups: orthologous genes in gene families that had significant expansion or contraction, and all other genes. Differences in genes under selection among climate zones and differences between the two groups of genes in each climate zone were tested using the Wilcoxon signed-rank test, with false discovery rate correction for *p*-values.

### GO enrichment in target genes

Potential functions of genes with significance in various analyses (gene family expansion and contraction, positive and negative selection) were examined using annotations from the *funannotate* pipeline (details in Additional File 2). First, gene names of all targets were summarised for each analysis. Any common genes were compared among genes under positively and negatively selection in each climate zone. GO enrichment was carried out using the R package *ClusterProfiler* v4.6.2 [133]. For this analysis, the background gene list consists of genes annotated for all samples (for gene family analysis), or genes annotated for all samples from particular climate zones (for selection analysis). Significant GO terms (*q*-value < 0.5) that are unique to, and shared between selection groups (1. alpine—positively selected, 2. alpine—negatively selected, 3. cold temperate—positively selected, 4. cold temperate—negatively selected, 5. Mediterranean—positively selected, 6. Mediterranean—negatively selected) were identified and summarised using the online tool *REVIGO* v1.8.1 [134]. The taxonomic relationship between *U. subpolyphylla* and *U. polyphylla* is not clear [54]. As gene gains and losses were identified across *U. subpolyphylla* samples compared to *U. polyphylla* samples, the above analyses were also carried out for these genes in gene families that expanded or contracted in all three *U. subpolyphylla* samples.

### Correlation between climate zone/bioclimatic variables and genomic features

We studied the potential influence of climate on genomic features focusing on three aspects: GC content, codon usage and amino acid usage. GC content for the first (GC1), second (GC2) and third (GC3) codon positions, as well as the relative synonymous codon usage (RSCU; the observed frequency divided by expected frequency of codons assuming equal expected frequency for all codons) were estimated using *BioKIT* v1.1.1 [135]. GC content of the first two codon positions (GC12) was calculated as the mean of GC1 and GC2. A neutrality plot of GC12 against GC3 was created to assess the relative effect of mutation bias and natural selection on codon usage: correlation between the two would indicate a stronger effect of mutation bias than that of natural selection. Correspondence analysis using RSCU of all samples was carried out using *R* v4.2.1 [136]. The number of each codon present in the annotated protein sequences, and the effective number of codons (ENC; as a proxy for codon bias) in each annotated gene of each sample was estimated using *CodonW* v1.4.2 (https://sourceforge.net/projects/codonw/), and the number of each amino acid was inferred from the results. Number of amino acid and codons were converted to percentage abundance afterwards. Genes with ENC ≤ 35 were considered ones with strong codon bias (strong usage preference). ENC was plotted against GC3 to assess the relative contributions of mutations and natural selection, in comparison to the curve of expected codon usage that is based on the formula: ENC = 2 + GC3 + 29/(GC3^2^ + (1 − GC3)^2^). Enrichment or depletion of amino acids between samples of the same species from different climate zones were tested using *Composition Profiler* v1.1 [137].

The above genomic features were investigated in two groups of genes: single-copy orthologues (identified by *OrthoFinder*), and all annotated genes of each sample. The use of single-copy orthologues is to detect any climate-related differences in genomic features in identical, conserved genes, whereas the use of all annotated genes is to detect the extent of climate-related effect in a genome-wide context (if any). The potential effect of climate on these genomic features in each group of genes was first studied by comparing GC content (GC12 and GC3), amino acid usage, codon usage and effective number of codons using samples grouped by their climate zone of collection. All features were averaged for each sample.

A second way of assessing the potential effect of climate was done using 11 bioclimatic variables associated with temperature (BIO1 – BIO11) from *WorldClim* v2.1 (spatial resolution of 30 s/~ 1 km^2^), which is a database of climatic data collected between 1970 and 2000 [138]. The bioclimatic variables associated with precipitation (BIO12 – BIO19) were excluded from analyses due to (1) most samples of the same species were collected from the same mountain at different elevations, but precipitation does not associate with elevation [139], and is more likely to be region-specific than site-specific; (2) *Umbilicaria* are rock-dwelling lichens in which the amount of precipitation is unrelated to moisture levels experienced by them, due to the lack of absorbent substrate. Bioclimatic variables were extracted using *QGIS* v3.4 (http://www.qgis.org). As many of these bioclimatic variables are correlated, a PCA was carried out to group correlated variables to the same PC. This is to avoid elevating the number of potential correlations with (seemingly) independent bioclimatic variables that actually describe similar climatic patterns. PCs that explain at least 5% of variance of data were retained (PC1 to PC3 in this case, see Results). For each selected PC, bioclimatic variables that contributed more than 10% were considered important factors: BIO1, 5, 10 and 11 for PC1; BIO2, 4, 6, 7 for PC2; BIO8, 9 for PC3 (see Results). Correlation between the three PCs and GC content (GC12 or GC3), amino acid usage, codon usage or effective number of codons were tested. *P*-values were corrected for false discovery rate (FDR) for analysis with amino acid usage (*N* = 20) and codon usage (*N* = 60) separately. *P*-values below 0.05 were considered significant. For significant correlations, relationship between bioclimatic variables with > 10% contribution to that PC and the target genomic feature was visualised as point graphs. Phylogenetic signals in these correlations were further tested using a Bayesian generalised linear multivariate multilevel model (details in Additional File 2).

### Intra-specific differences in methylation patterns

We tested for potential differences in the level of methylation between samples of the same species collected from different climate zones. Using the 18 samples from eight species that have at least two samples with kinetic information supplied (Additional File 1: Table S1), we first predicted 5-methylcytosine (5mC) of each CpG site using the package *pbjasmine* v2.0.0 (PacificBiosciences). The *pbmm2* v1.13.1 package (PacificBiosciences) was then used to align the resulting bam files to the *U. pustulata* (TBG2345) reference genome. The *–preset* flag was set to *SUBREAD* for the two *U. phaea* samples. Duplicates were removed from the mapped files using the *MarkDuplicates* v3.1.1 function of the *picard* package [140]. Methylation was called using the *aligned_bam_to_cpg_scores* script of *pb-CpG-tools* v2.3.2 (PacificBiosciences), using the *count* pileup method and the *U. pustulata* (TBG2345) reference genome. Methylated sites with coverage less than 10 and modification score less than 50 were removed. Percentage of methylated sites was calculated for 1 Mb non-overlapping windows. Differences in percentage of methylated sites between samples from different climate zones (same species) were tested using the Kruskal–Wallis test.

## Supplementary Information

Additional file 1: Table S1-S27. Table S1 – Sample details. Table S2 – Gene products in significantly changed gene families. Table S3 – GO enrichment. Table S4 – Number of genes under selection. Table S5-S10 GO enrichment. Table S11 – Unique GO terms under selection. Table S12 – Shared GO terms among zones. Table S13 – PCA loading values. Table S14 – Contributions of BIOCLIM variables to PCs. Table S15 – Correlation between genomic features and BIOCLIM variables. Table S16 – GO terms. Table S17 – InterPro families. Table S18 – COG. Table S19 – CAZymes. Table S20-S25 – InterPro families. Table S26 – InterPro families. Table S27 – Genes under strong codon bias. Table S28 – Pagel’s λ.

Addition File 2: Supplementary information and Figure S1-S15. Figure S1– Phylogram and chronogram. Figure S2 – Genome telomere ends. Figure S3 – Secondary metabolism gene trees. Figure S4-S5 – Amino acid differences among climate zones. Figure S6 – PCA statistics. Figure S7 – GC differences among climate zones. Figure S8 – Number of ENC in climate zones. Figure S9 – CAZymes in climate zones. Figure S10 – Correspondence analysis of RSCU. Figure S11 – BIOCLIM distribution in climate zones. Figure S12 – Methylation patterns. Figure S13 – Annotations of gene family changes in *U. subpolyphylla*. Figure S14 – Gene outliers in difference analyses. Figure S15 – ENC VS GC3.

## Data Availability

Metagenomic and metatranscriptomic raw reads have been submitted to the Sequence Read Archive (SRA) database under GenBank BioProject PRJNA1255408, except RNA sequences from the two *U. pustulata* samples which are available under the ENA BioProject PRJEB72275. For DNA (all GenBank): Lichen metagenome BioSample accessions are SAMN17491176 – SAMN17491177 and SAMN48142972 - SAMN48143006. The mycobionts have the BioSample accessions SAMN48289772-SAMN48289798 and are linked to their corresponding lichen metagenome BioSamples. Lichen metagenomic reads are deposited under the accessions SRR33313076 – SRR33313109 and SRR13514086 – SRR13514087. The assembled mycobiont genomes and their annotations are deposited under the accessions JBNORA000000000-JBNOSA000000000. For RNA: Lichen metagenome BioSample accessions are SAMN48142999 – SAMN48143006 (GenBank), SAMEA115171027 (ENA) and SAMEA115171030 (ENA). Lichen metatranscriptomic reads are deposited under the GenBank accessions SRR33313075, SRR33313077 - SRR33313081, SRR33313096 - SRR33313097, and ENA accessions ERR13033436 and ERR13033439. All details can be found in Table S1.

## Abbreviations

5mC: 5 methylcytosine
ANOVA: Analysis of variance
BUSCO: Benchmarking Universal Single-Copy Orthologs
CAFE: Computational Analysis of gene Family Evolution
CAZyme: Carbohydrate-Active enZYmes
DNA: Deoxyribonucleic acid
ENC: Effective number of codons
FDR: False discovery rate
GO: Gene ontology
NCBI: National Center for Biotechnology Information
PC: Principal component
PCA: Principal component analysis
PKS: Polyketide synthases
REVIGO: Reduce & Visualise Gene Ontology
RNA: Ribonucleic acid
RSCU: Relative synonymous codon usage
STAG: Species Tree Inference from All Genes
tRNA: Transfer ribonucleic acid
UV: Ultraviolet

## References

[1] Mpakairi KS, Ndaimani H, Tagwireyi P, Zvidzai M, Madiri TH. Futuristic climate change scenario predicts a shrinking habitat for the African elephant (Loxodonta africana): evidence from Hwange National Park, Zimbabwe. Eur J Wildl Res. 2020;66:1–10. 10.1007/s10344-019-1327-x.

[2] Soroye P, Newbold T, Kerr J. Climate change contributes to widespread declines among bumble bees across continents. Science. 2020;367:685–8.10.1126/science.aax8591 32029628 10.1126/science.aax8591

[3] Pinsky ML, Selden RL, Kitchel ZJ. Climate-driven shifts in marine species ranges: scaling from organisms to communities. Ann Rev Mar Sci. 2020;12:153–79. 10.1146/annurev-marine-010419-010916. 31505130 10.1146/annurev-marine-010419-010916

[4] Román-Palacios C, Wiens JJ. Recent responses to climate change reveal the drivers of species extinction and survival. Proc Natl Acad Sci U S A. 2020;117:4211–7. 10.1073/pnas.1913007117. 32041877 10.1073/pnas.1913007117 PMC7049143

[5] Morgan R, Finnøen MH, Jensen H, Pélabon C, Jutfelt F. Low potential for evolutionary rescue from climate change in a tropical fish. Proc Natl Acad Sci U S A. 2020;117:33365–72. 10.1073/pnas.2011419117. 33318195 10.1073/pnas.2011419117 PMC7776906

[6] Brady SP, Bolnick DI, Angert AL, Gonzalez A, Barrett RDH, Crispo E, et al. Causes of maladaptation. Evol Appl. 2019;12:1229–42. 10.1111/eva.12844. 31417611 10.1111/eva.12844 PMC6691215

[7] Bradshaw WE, Holzapfel CM. Evolutionary response to rapid climate change. Science. 2006;312:1477–8. 10.1126/science.1127000 16763134 10.1126/science.1127000

[8] Franks SJ, Hamann E, Weis AE. Using the resurrection approach to understand contemporary evolution in changing environments. Evol Appl. 2018;11:17–28. 10.1111/eva.12528. 29302269 10.1111/eva.12528 PMC5748528

[9] Fraser DJ, Walker L, Yates MC, Marin K, Wood JLA, Bernos TA, et al. Population correlates of rapid captive-induced maladaptation in a wild fish. Evol Appl. 2019;12:1305–17. 10.1111/eva.12649. 31417616 10.1111/eva.12649 PMC6691219

[10] Walters RJ, Berger D. Implications of existing local (mal)adaptations for ecological forecasting under environmental change. Evol Appl. 2019;12:1487–502. 10.1111/eva.12840. 31417629 10.1111/eva.12840 PMC6691230

[11] Zeldovich KB, Berezovsky IN, Shakhnovich EI. Protein and DNA Sequence Determinants of Thermophilic Adaptation. PLOS Comput Biol. 2007;3:e5. 10.1371/journal.pcbi.0030005 17222055 10.1371/journal.pcbi.0030005 PMC1769408

[12] Liu Q, Song WZ, Zhou YG, Dong XZ, Xin YH. Phenotypic divergence of thermotolerance: Molecular basis and cold adaptive evolution related to intrinsic DNA flexibility of glacier-inhabiting *Cryobacterium* strains. Environ Microbiol. 2020;22:1409–20. 10.1111/1462-2920.14957. 32090405 10.1111/1462-2920.14957

[13] Basak S, Ghosh TC. On the origin of genomic adaptation at high temperature for prokaryotic organisms. Biochem Biophys Res Commun. 2005;330:629–32. 10.1016/j.bbrc.2005.02.134. 15809043 10.1016/j.bbrc.2005.02.134

[14] Felipe Benites L, Stephens TG, Van Etten J, James T, Christian WC, Barry K, et al. Hot springs viruses at Yellowstone National Park have ancient origins and are adapted to thermophilic hosts. Commun Biol 2024 71. 2024;7:1–12.10.1038/s42003-024-05931-1 10.1038/s42003-024-05931-1 PMC11003980 38594478

[15] Lobry JR, Necşulea A. Synonymous codon usage and its potential link with optimal growth temperature in prokaryotes. Gene. 2006;385:128–36.10.1016/j.gene.2006.05.033 16989961 10.1016/j.gene.2006.05.033

[16] Raymond-Bouchard I, Goordial J, Zolotarov Y, Ronholm J, Stromvik M, Bakermans C, et al. Conserved genomic and amino acid traits of cold adaptation in subzero-growing Arctic permafrost bacteria. FEMS Microbiol Ecol. 2018;94: fiy023. 10.1093/femsec/fiy023. 10.1093/femsec/fiy023 29528411

[17] Panda A, Tuller T. Determinants of associations between codon and amino acid usage patterns of microbial communities and the environment inferred based on a cross-biome metagenomic analysis. npj Biofilms Microbiomes. 2023;9:1–18. 10.1038/s41522-023-00372-w 10.1038/s41522-023-00372-w PMC9873608 36693851

[18] Higgs PG, Ran W. Coevolution of codon usage and tRNA genes leads to alternative stable states of biased codon usage. Mol Biol Evol. 2008;25:2279–91. 10.1093/molbev/msn173. 18687657 10.1093/molbev/msn173

[19] Shah P, Gilchrist MA. Effect of Correlated tRNA Abundances on Translation Errors and Evolution of Codon Usage Bias. PLOS Genet. 2010;6:e1001128.10.1371/journal.pgen.1001128 20862306 10.1371/journal.pgen.1001128 PMC2940732

[20] Su Y, Jiang X, Wu W, Wang M, Imran Hamid M, Xiang M, et al. Genomic, transcriptomic, and proteomic analysis provide insights into the cold adaptation mechanism of the obligate psychrophilic fungus *Mrakia psychrophila*. G3 Genes, Genomes, Genet. 2016;6:3603–13. 10.1534/g3.116.033308 10.1534/g3.116.033308 PMC5100859 27633791

[21] Sælensminde G, Halskau Ø, Jonassen I. Amino acid contacts in proteins adapted to different temperatures: Hydrophobic interactions and surface charges play a key role. Extremophiles. 2009;13:11–20.10.1007/s00792-008-0192-4 18825305 10.1007/s00792-008-0192-4

[22] Tronelli D, Maugini E, Bossa F, Pascarella S. Structural adaptation to low temperatures − analysis of the subunit interface of oligomeric psychrophilic enzymes. FEBS J. 2007;274:4595–608. 10.1111/j.1742-4658.2007.05988.x. 17697122 10.1111/j.1742-4658.2007.05988.x

[23] Georlette D, Blaise V, Collins T, D’Amico S, Gratia E, Hoyoux A, et al. Some like it cold: biocatalysis at low temperatures. FEMS Microbiol Rev. 2004;28:25–42.10.1016/j.femsre.2003.07.003 14975528 10.1016/j.femsre.2003.07.003

[24] Saunders NFW, Thomas T, Curmi PMG, Mattick JS, Kuczek E, Slade R, et al. Mechanisms of thermal adaptation revealed from the genomes of the Antarctic Archaea *Methanogenium frigidum* and *Methanococcoides burtonii*. Genome Res. 2003;13:1580–8. 10.1101/gr.1180903. 12805271 10.1101/gr.1180903 PMC403754

[25] Gerday C, Aittaleb M, Bentahir M, Chessa JP, Claverie P, Collins T, et al. Cold-adapted enzymes: from fundamentals to biotechnology. Trends Biotechnol. 2000;18:103–7. 10.1016/s0167-7799(99)01413-4. 10675897 10.1016/s0167-7799(99)01413-4

[26] Metpally RPR, Reddy BVB. Comparative proteome analysis of psychrophilic versus mesophilic bacterial species: Insights into the molecular basis of cold adaptation of proteins. BMC Genomics. 2009;10:1–10. 10.1186/1471-2164-10-11 19133128 10.1186/1471-2164-10-11 PMC2653534

[27] Nizovoy P, Bellora N, Haridas S, Sun H, Daum C, Barry K, et al. Unique genomic traits for cold adaptation in Naganishia vishniacii, a polyextremophile yeast isolated from Antarctica. FEMS Yeast Res. 2021;21:56. 10.1093/femsyr/foaa056. 10.1093/femsyr/foaa056 33232451

[28] Hu X, Zhang YJ, Xiao GH, Zheng P, Xia YL, Zhang XY, et al. Genome survey uncovers the secrets of sex and lifestyle in caterpillar fungus. Chin Sci Bull. 2013;58:2846–54. 10.1007/s11434-013-5929-5.

[29] Tsuji M, Fujiu S, Xiao N, Hanada Y, Kudoh S, Kondo H, et al. Cold adaptation of fungi obtained from soil and lake sediment in the Skarvsnes ice-free area, Antarctica. FEMS Microbiol Lett. 2013;346:121–30. 10.1111/1574-6968.12217 23862768 10.1111/1574-6968.12217

[30] Tyagi S, Kabade PG, Gnanapragasam N, Singh UM, Gurjar AKS, Rai A, et al. Codon usage provide insights into the adaptation of rice genes under stress condition. Int J Mol Sci. 2023;24: 1098. 10.3390/ijms24021098. 36674611 10.3390/ijms24021098 PMC9861248

[31] Katz RW, Brown BG. Extreme events in a changing climate: Variability is more important than averages. Clim Change. 1992;21:289–302.

[32] Ikeda DH, Max TL, Allan GJ, Lau MK, Shuster SM, Whitham TG. Genetically informed ecological niche models improve climate change predictions. Glob Chang Biol. 2017;23:164–76. 10.1111/gcb.13470. 27543682 10.1111/gcb.13470

[33] Sheth SN, Angert AL. The evolution of environmental tolerance nad range size: a comparison of geographically restricted and widespread Mimulus. Evolution. 2014;68:2917–31. 10.1111/evo.12494. 10.1111/evo.12494 25066881

[34] 014;68:2917–31. DOI: 10.1111/evo.12494 34. Schwarzwald K, Lenssen N. The importance of internal climate variability in climate impact projections. Proc Natl Acad Sci. 2022;119:e2208095119.10.1073/pnas.2208095119 36215470 10.1073/pnas.2208095119 PMC9586330

[35] Capblancq T, Fitzpatrick MC, Bay RA, Exposito-Alonso M, Keller SR. Genomic prediction of (mal)adaptation across current and future climatic landscapes. Annu Rev Ecol Evol Syst. 2020;51(1):245–69. 10.1146/annurev-ecolsys-020720-042553.

[36] Alberto FJ, Aitken SN, Alía R, González-Martínez SC, Hänninen H, Kremer A, et al. Potential for evolutionary responses to climate change – evidence from tree populations. Glob Chang Biol. 2013;19:1645–61. 10.1111/gcb.12181. 23505261 10.1111/gcb.12181 PMC3664019

[37] Benito Garzón M, Robson TM, Hampe A. ΔTraitSDMs: species distribution models that account for local adaptation and phenotypic plasticity. New Phytol. 2019;222:1757–65. 10.1111/nph.15716. 30697749 10.1111/nph.15716

[38] Vasseur DA, DeLong JP, Gilbert B, Greig HS, Harley CDG, McCann KS, et al. Increased temperature variation poses a greater risk to species than climate warming. Proc R Soc Lond B Biol Sci. 2014;281: 20132612. 10.1098/rspb.2013.2612. 10.1098/rspb.2013.2612 PMC3924069 24478296

[39] Hasegawa T, Sakurai G, Fujimori S, Takahashi K, Hijioka Y, Masui T. Extreme climate events increase risk of global food insecurity and adaptation needs. Nat Food. 2021;2:587–95.10.1038/s43016-021-00335-4 37118168 10.1038/s43016-021-00335-4

[40] Murdock CC, Paaijmans KP, Cox-Foster D, Read AF, Thomas MB. Rethinking vector immunology: the role of environmental temperature in shaping resistance. Nat Rev Microbiol. 2012;10:869–76. 10.1038/nrmicro2900 23147703 10.1038/nrmicro2900 PMC4142813

[41] . Herren CM, Baym M. Decreased thermal niche breadth as a trade-off of antibiotic resistance. ISME J. 2022;16:1843–52. 10.1038/s41396-022-01235-6 35422477 10.1038/s41396-022-01235-6 PMC9213455

[42] Dewenter BS, Shah AA, Hughes J, Poff NLR, Thompson R, Kefford BJ. The thermal breadth of temperate and tropical freshwater insects supports the climate variability hypothesis. Ecol Evol. 2024;14: e10937. 10.1002/ece3.10937. 38405410 10.1002/ece3.10937 PMC10891360

[43] Strader RN, Dowd SC, Blawas C, Mahoney RD, Patetta NC, Leslie J, et al. Climate variability hypothesis is partially supported in thermal limits of juvenile Northwest Atlantic coastal fishes. J Fish Biol. 2023;103:1452–62. 10.1111/jfb.15533. 37650861 10.1111/jfb.15533

[44] Diamond SE. Evolutionary potential of upper thermal tolerance: biogeographic patterns and expectations under climate change. Ann N Y Acad Sci. 2017;1389:5–19. 10.1111/nyas.13223. 27706832 10.1111/nyas.13223

[45] Dowd WW, King FA, Denny MW, Podrabsky JE, Stillman JH, Tomanek L. Thermal variation, thermal extremes and the physiological performance of individuals. J Exp Biol. 2015;218:1956–67. 10.1242/jeb.114926 26085672 10.1242/jeb.114926

[46] Woods HA, Dillon ME, Pincebourde S. The roles of microclimatic diversity and of behavior in mediating the responses of ectotherms to climate change. J Therm Biol. 2015;54:86–97. 10.1016/j.jtherbio.2014.10.002. 26615730 10.1016/j.jtherbio.2014.10.002

[47] Pincebourde S, Casas J. Narrow safety margin in the phyllosphere during thermal extremes. Proc Natl Acad Sci U S A. 2019;116:5588–96. 10.1073/pnas.1815828116. 30782803 10.1073/pnas.1815828116 PMC6431205

[48] Colinet H, Sinclair BJ, Vernon P, Renault D. Insects in fluctuating thermal environments. Annu Rev Entomol. 2015;60 Volume 60, 2015:123–40. 10.1146/annurev-ento-010814-021017 10.1146/annurev-ento-010814-021017 25341105

[49] Dillon ME, Lozier JD. Adaptation to the abiotic environment in insects: the influence of variability on ecophysiology and evolutionary genomics. Curr Opin Insect Sci. 2019;36:131–9. 10.1016/j.cois.2019.09.003 31698151 10.1016/j.cois.2019.09.003

[50] dos Santos EV, Martinez PA, Souza G, Jacobina UP. Genome size drives ecological breadth in Pomacentridae reef fishes. J Exp Mar Bio Ecol. 2021;540: 151544. 10.1016/j.jembe.2021.151544.

[51] Opulente DA, LaBella AL, Harrison MC, Wolters JF, Liu C, Li Y, et al. Genomic factors shape carbon and nitrogen metabolic niche breadth across Saccharomycotina yeasts. Science. 2024;384:eadj4503.10.1126/science.adj4503 38662846 10.1126/science.adj4503 PMC11298794

[52] Stillman JH, Tagmount A. Seasonal and latitudinal acclimatization of cardiac transcriptome responses to thermal stress in porcelain crabs, *Petrolisthes cinctipes*. Mol Ecol. 2009;18:4206–26. 10.1111/j.1365-294X.2009.04354.x. 19765222 10.1111/j.1365-294X.2009.04354.x

[53] Rain-Franco A, Mouquet N, Gougat-Barbera C, Bouvier T, Beier S. Niche breadth affects bacterial transcription patterns along a salinity gradient. Mol Ecol. 2022;31:1216–33. 10.1111/mec.16316 34878694 10.1111/mec.16316

[54] Davydov EA, Peršoh D, Rambold G. Umbilicariaceae (lichenized Ascomycota) – trait evolution and a new generic concept. Taxon. 2019;66:1282–303. 10.12705/666.2.

[55] Kappen L. Plant Activity under Snow and Ice, with Particular Reference to Lichens. Arctic. 1993;46:297–302.

[56] Kappen L, Schroeter B, Scheidegger C, Sommerkorn M, Hestmark G. Cold resistance and metabolic activity of lichens below 0°c. Adv Space Res. 1996;18:119–28. 10.1016/0273-1177(96)00007-5.11538952

[57] Dal Grande F, Sharma R, Meiser A, Rolshausen G, Büdel B, Mishra B, et al. Adaptive differentiation coincides with local bioclimatic conditions along an elevational cline in populations of a lichen-forming fungus. BMC Evol Biol. 2017;17:93. 10.1186/s12862-017-0929-8 28359299 10.1186/s12862-017-0929-8 PMC5374679

[58] Rolshausen G, Hallman U, Grande FD, Otte J, Knudsen K, Schmitt I. Expanding the mutualistic niche: parallel symbiont turnover along climatic gradients. Proc R Soc B-Biol Sci. 2020;287: 20192311. 10.1098/rspb.2019.2311. 10.1098/rspb.2019.2311 PMC7209069 32228406

[59] Wong ELY, Valim H, Schmitt I. Genome‐wide differentiation corresponds to climatic niches in two species of lichen‐forming fungi. Environ Microbiol. 2024;26: e16703. 10.1111/1462-2920.16703. 39388227 10.1111/1462-2920.16703

[60] Valim HF, Grande FD, Wong ELY, Schmitt I. Circadian clock- and temperature-associated genes contribute to overall genomic differentiation along elevation in lichenized fungi. Mol Ecol. 2024;33: e17252. 10.1111/mec.17252. 38146927 10.1111/mec.17252

[61] Merges D, Dal Grande F, Valim H, Singh G, Schmitt I. Gene abundance linked to climate zone: Parallel evolution of gene content along elevation gradients in lichenized fungi. Front Microbiol. 2023;14:1097787.10.3389/fmicb.2023.1097787 37032854 10.3389/fmicb.2023.1097787 PMC10073550

[62] Dal Grande F, Jamilloux V, Choisne N, Calchera A, Rolshausen G, Petersen M, et al. Transposable Elements in the Genome of the Lichen-Forming Fungus Umbilicaria pustulata and Their Distribution in Different Climate Zones along Elevation. Biology (Basel). 2021;11:24. 10.3390/biology11010024. 10.3390/biology11010024 PMC8773270 35053022

[63] Singh G, Calchera A, Schulz M, Drechsler M, Bode HB, Schmitt I, et al. Climate‐specific biosynthetic gene clusters in populations of a lichen‐forming fungus. Environ Microbiol. 2021;23:4260–75. 10.1111/1462-2920.15605. 34097344 10.1111/1462-2920.15605

[64] Rolshausen G, Dal Grande F, Otte J, Schmitt Imke. Lichen holobionts show compositional structure along elevation. Mol Ecol. 2022;32:6619–30. 10.1111/mec.16471. 35398946 10.1111/mec.16471

[65] Merges D, Dal Grande F, Greve C, Otte J, Schmitt I. Virus diversity in metagenomes of a lichen symbiosis (Umbilicaria phaea): complete viral genomes, putative hosts and elevational distributions. Environ Microbiol. 2021;23:6637–50. 10.1111/1462-2920.15802. 10.1111/1462-2920.15802 34697892

[66] Vargas Castillo R, Beck A. Photobiont selectivity and specificity in *Caloplaca* species in a fog-induced community in the Atacama Desert, northern Chile. Fungal Biol. 2012;116:665–76. 10.1016/j.funbio.2012.04.001. 22658312 10.1016/j.funbio.2012.04.001

[67] Werth S, Sork VL. Ecological specialization in *Trebouxia* (Trebouxiophyceae) photobionts of *Ramalina menziesii* (Ramalinaceae) across six range-covering ecoregions of western North America. Am J Bot. 2014;101(7):1127–40. 10.3732/ajb.1400025. 25016008 10.3732/ajb.1400025

[68] f western North America. Am J Bot. 2014;101:1127–40. DOI: 10.3732/ajb.1400025 68. Muggia L, Pérez-Ortega S, Kopun T, Zellnig G, Grube M. Photobiont selectivity leads to ecological tolerance and evolutionary divergence in a polymorphic complex of lichenized fungi. Ann Bot. 2014;114:463–75. 10.1093/aob/mcu146 25096324 10.1093/aob/mcu146 PMC4204673

[69] Rolshausen G, Dal Grande F, Sadowska-Deś AD, Otte J, Schmitt I. Quantifying the climatic niche of symbiont partners in a lichen symbiosis indicates mutualist-mediated niche expansions. Ecography. 2018;41(8):1380–92. 10.1111/ecog.03457.

[70] Felsenstein J. Phylogenies and the comparative method. Am Nat. 1985;125:1–15.

[71] Chen L, Li Y, Zhang Q, Wang D, Akhberdi O, Wei D, et al. Improved pestalotiollide B production by deleting competing polyketide synthase genes in *Pestalotiopsis microspora*. J Ind Microbiol Biotechnol. 2017;44:237–46. 10.1007/s10295-016-1882-z. 28005187 10.1007/s10295-016-1882-z

[72] Liu Y, Chen L, Xie Q, Yu X, Duan A, Lin Y, et al. A gene cluster for the biosynthesis of dibenzodioxocinons in the endophyte *Pestalotiopsis microspora*, a taxol producer. J Microbiol Biotechnol. 2019;29:1570–9. 10.4014/jmb.1905.05051. 31474098 10.4014/jmb.1905.05051

[73] Li S, Wang D, He J, Liao C, Zuo Z, Li S, et al. Thermophilic fungus uses anthraquinones to modulate ferrous excretion, sterol-mediated endocytosis, and iron storage in response to cold stress. Microb Biotechnol. 2024;17: e70002. 10.1111/1751-7915.70002. 39212141 10.1111/1751-7915.70002 PMC11362841

[74] Khan MI, Bashir N, Pandith SA, Patil SS, Pable AA, Shah MA, et al. Low temperature stress modulates the biochemical, metabolic, and molecular behavior of the Trans-Himalayan medicinal herb *Rheum spiciforme* Royle (spiked rhubarb). Ind Crop Prod. 2023;193: 116154. 10.1016/j.indcrop.2022.116154.

[75] Oulmouden A, Karst F. Nucleotide sequence of the ERG12 gene of *Saccharomyces cerevisiae* encoding mevalonate kinase. Curr Genet. 1991;19:9–14. 10.1007/BF00362081. 1645230 10.1007/BF00362081

[76] . Xu YJ, Singh A, Alter GM. Hydroxyurea induces cytokinesis arrest in cells expressing a mutated sterol-14α-demethylase in the ergosterol biosynthesis pathway. Genetics. 2016;204:959–73. 10.1534/genetics.116.191536 27585850 10.1534/genetics.116.191536 PMC5105871

[77] Chen Q, Steinhauer L, Hammerlindl J, Keller W, Zou J. Biosynthesis of phytosterol esters: identification of a sterol O-acyltransferase in Arabidopsis. Plant Physiol. 2007;145:974–84. 10.1104/pp.107.106278. 17885082 10.1104/pp.107.106278 PMC2048776

[78] Grabińska K, Palamarczyk G. Dolichol biosynthesis in the yeast Saccharomyces cerevisiae: an insight into the regulatory role of farnesyl diphosphate synthase. FEMS Yeast Res. 2002;2:259–65. 10.1016/S1567-1356(02)00110-1 12702274 10.1016/S1567-1356(02)00110-1

[79] Jordá T, Puig S. Regulation of ergosterol biosynthesis in *Saccharomyces cerevisiae*. Genes. 2020;11: 795. 10.3390/genes11070795. 32679672 10.3390/genes11070795 PMC7397035

[80] Liebl M, Huber L, Elsaman H, Merschak P, Wagener J, Gsaller F, et al. Quantifying Isoprenoids in the Ergosterol Biosynthesis by Gas Chromatography–Mass Spectrometry. J Fungi. 2023;9:768. 10.3390/jof9070768 10.3390/jof9070768 PMC10381423 37504756

[81] Weete J. Lipid biochemistry of fungi and other organisms. Springer Science & Business Media; 2012. 10.1007/978-1-4757-0064-0

[82] Kohlhase M, Pohl P. Saturated and unsaturated sterols of nitrogen-fixing blue-green algae (cyanobacteria). Phytochemistry. 1988;27:1735–40.10.1016/0031-9422(88)80434-5

[83] Stewart W. Algal physiology and biochemistry. Univ of California Press; 1974.

[84] Wang T, Bengtsson G, Kärnefelt I, Björn LO. Provitamins and vitamins D2 and D3 in *Cladina* spp. over a latitudinal gradient: possible correlation with UV levels. J Photochem Photobiol B Biol. 2001;62:118–22. 10.1016/s1011-1344(01)00160-9. 10.1016/s1011-1344(01)00160-9 11693362

[85] Rao Koyyalamudi S, Jeong S, Song C, Yip Cho K, Pang G. Vitamin D2 formation and bioavailability from Agaricus bisporus button mushrooms treated with ultraviolet irradiation. J Agric Food Chem. 2009;57:3351–5. 10.1021/jf803908q 19281276 10.1021/jf803908q

[86] Bhattacharya S, Esquivel BD, White TC. Overexpression or deletion of ergosterol biosynthesis genes alters doubling time, response to stress agents, and drug susceptibility in Saccharomyces cerevisiae. MBio. 2018. 10.1128/mBio.01291-18. 30042199 10.1128/mBio.01291-18 PMC6058291

[87] Blosser SJ, Merriman B, Grahl N, Chung D, Cramer RA. Two C4-sterol methyl oxidases (Erg25) catalyse ergosterol intermediate demethylation and impact environmental stress adaptation in *Aspergillus fumigatus*. Microbiology. 2014;160:2492–506. 10.1099/mic.0.080440-0. 25107308 10.1099/mic.0.080440-0 PMC4219106

[88] Dupont S, Lemetais G, Ferreira T, Cayot P, Gervais P, Beney L. Ergosterol biosyntehsis: a fungal pathway for life on land? Evolution. 2012;66:2961–8. 10.1111/j.1558-5646.2012.01667.x 22946816 10.1111/j.1558-5646.2012.01667.x

[89] Yakobov N, Fischer F, Mahmoudi N, Saga Y, Grube CD, Roy H, et al. RNA-dependent sterol aspartylation in fungi. Proc Natl Acad Sci. 2020;117:14948–5710.1073/pnas.2003266117 32541034 10.1073/pnas.2003266117 PMC7334510

[90] Chakraborty A, Fernando LD, Fang W, Dickwella Widanage MC, Wei P, Jin C, et al. A molecular vision of fungal cell wall organization by functional genomics and solid-state NMR. Nat Commun. 2021;12:1–12. 10.1038/s41467-021-26749-z 34732740 10.1038/s41467-021-26749-z PMC8566572

[91] Krause F, Scheckhuber CQ, Werner A, Rexroth S, Reifschneider NH, Dencher NA, et al. Supramolecular organization of cytochrome c oxidase- and alternative oxidase-dependent respiratory chains in the filamentous fungus *Podospora anserina*. J Biol Chem. 2004;279:26453–61. 10.1074/jbc.M402756200. 15044453 10.1074/jbc.M402756200

[92] Cordero RJB, Casadevall A. Functions of fungal melanin beyond virulence. Fungal Biol Rev. 2017;31:99–112.10.1016/j.fbr.2016.12.003 31649746 10.1016/j.fbr.2016.12.003 PMC6812541

[93] Camacho E, Vij R, Chrissian C, Prados-Rosales R, Gil D, O’Meally RN, et al. The structural unit of melanin in the cell wall of the fungal pathogen *Cryptococcus neoformans*. J Biol Chem. 2019;294:10471–89. 10.1074/jbc.RA119.008684. 31118223 10.1074/jbc.RA119.008684 PMC6615676

[94] Fernando LD, Pérez-Llano Y, Dickwella Widanage MC, Jacob A, Martínez-Ávila L, Lipton AS, et al. Structural adaptation of fungal cell wall in hypersaline environment. Nat Commun. 2023;14:1–13.10.1038/s41467-023-42693-6 37925437 10.1038/s41467-023-42693-6 PMC10625518

[95] Byun MY, Cui LH, Lee A, Oh HG, Yoo YH, Lee J, et al. Abiotic stress-induced actin-depolymerizing factor 3 from *Deschampsia antarctica* enhanced cold tolerance when constitutively expressed in rice. Front Plant Sci. 2021;12: 734500. 10.3389/fpls.2021.734500. 34650582 10.3389/fpls.2021.734500 PMC8506025

[96] Sun Y, Shi M, Wang D, Gong Y, Sha Q, Lv P, et al. Research progress on the roles of actin-depolymerizing factor in plant stress responses. Front Plant Sci. 2023;14:1278311. 10.3389/fpls.2023.1278311 38034575 10.3389/fpls.2023.1278311 PMC10687421

[97] Abu Bakar N, Lau BYC, González-Aravena M, Smykla J, Krzewicka B, Karsani SA, et al. Geographical diversity of proteomic responses to cold stress in the fungal genus *Pseudogymnoascus*. Microb Ecol. 2024;87:1–25. 10.1007/s00248-023-02311-w. 10.1007/s00248-023-02311-w PMC10703952 38060022

[98] Danyluk J, Carpentier E, Sarhan F. Identification and characterization of a low temperature regulated gene encoding an actin-binding protein from wheat. FEBS Lett. 1996;389:324–7. 10.1016/0014-5793(96)00599-6 8766725 10.1016/0014-5793(96)00599-6

[99] Örvar BL, Sangwan V, Omann F, Dhindsa RS. Early steps in cold sensing by plant cells: the role of actin cytoskeleton and membrane fluidity. Plant J. 2000;23:785–94. 10.1046/j.1365-313x.2000.00845.x 10998189 10.1046/j.1365-313x.2000.00845.x

[100] Sangwan V, Örvar BL, Beyerly J, Hirt H, Dhindsa Rajinder S. Opposite changes in membrane fluidity mimic cold and heat stress activation of distinct plant MAP kinase pathways. Plant J. 2002;31:629–38. 10.1046/j.1365-313x.2000.00845.x 12207652 10.1046/j.1365-313x.2002.01384.x

[101] Fujita S, Pytela J, Hotta T, Kato T, Hamada T, Akamatsu R, et al. An atypical tubulin kinase mediates stress-induced microtubule depolymerization in Arabidopsis. Curr Biol. 2013;23:1969–78. 10.1016/j.cub.2013.08.006. 24120637 10.1016/j.cub.2013.08.006

[102] Shukla N, Osmani AH, Osmani SA. Microtubules are reversibly depolymerized in response to changing gaseous microenvironments within *Aspergillus nidulans* biofilms. Mol Biol Cell. 2017;28:634–44. 10.1091/mbc.E16-10-0750. 28057761 10.1091/mbc.E16-10-0750 PMC5328622

[103] Nick P. Microtubules, signalling and abiotic stress. Plant J. 2013;75:309–23.10.1111/tpj.12102 23311499 10.1111/tpj.12102

[104] Abdrakhamanova A, Wang QY, Khokhlova L, Nick P. Is Microtubule Disassembly a Trigger for Cold Acclimation? Plant Cell Physiol. 2003;44:676–86. 10.1093/pcp/pcg097 12881495 10.1093/pcp/pcg097

[105] Zhang X, Chen X, Jiang J, Yu M, Yin Y, Ma Z. The tubulin cofactor a is involved in hyphal growth, conidiation and cold sensitivity in *Fusarium asiaticum*. BMC Microbiol. 2015;15:1–13. 10.1186/s12866-015-0374-z. 25886735 10.1186/s12866-015-0374-z PMC4342098

[106] Ma H, Liu M. The microtubule cytoskeleton acts as a sensor for stress response signaling in plants. Mol Biol Rep. 2019;46:5603–8. 10.1007/s11033-019-04872-x 31098806 10.1007/s11033-019-04872-x

[107] . Nyporko AY, Demchuk ON, Blume YB. Cold adaptation of plant microtubules: structural interpretation of primary sequence changes in a highly conserved region of α-tubulin. Cell Biol Int. 2003;27:241–3. 10.1016/s1065-6995(02)00342-6 12681322 10.1016/s1065-6995(02)00342-6

[108] Medina A, Schmidt-Heydt M, Rodríguez A, Parra R, Geisen R, Magan N. Impacts of environmental stress on growth, secondary metabolite biosynthetic gene clusters and metabolite production of xerotolerant/xerophilic fungi. Curr Genet. 2015;61:325–34.10.1007/s00294-014-0455-9 25381155 10.1007/s00294-014-0455-9

[109] Broberg M, Dubey M, Sun MH, Ihrmark K, Schroers HJ, Li SD, et al. Out in the cold: identification of genomic regions associated with cold tolerance in the biocontrol fungus *Clonostachys rosea* through genome-wide association mapping. Front Microbiol. 2018. 10.3389/fmicb.2018.02844. 30524411 10.3389/fmicb.2018.02844 PMC6262169

[110] Lenardon MD, Lesiak I, Munro CA, Gow NAR. Dissection of the *Candida albicans* class i chitin synthase promoters. Mol Genet Genomics. 2009;281:459–71. 10.1007/s00438-009-0423-0. 19153767 10.1007/s00438-009-0423-0 PMC3468743

[111] Liu R, Xu C, Zhang Q, Wang S, Fang W. Evolution of the chitin synthase gene family correlates with fungal morphogenesis and adaption to ecological niches. Sci Rep. 2017;7:1–12.10.1038/srep44527 28300148 10.1038/srep44527 PMC5353729

[112] Nakamura T, Ishikawa M, Nakatani H, Oda A. Characterization of Cold-Responsive Extracellular Chitinase in Bromegrass Cell Cultures and Its Relationship to Antifreeze Activity. Plant Physiol. 2008;147:391–401. 10.1104/pp.106.081497 18359848 10.1104/pp.106.081497 PMC2330313

[113] Li XC, Peris D, Hittinger CT, Sia EA, Fay JC. Mitochondria-encoded genes contribute to evolution of heat and cold tolerance in yeast. Sci Adv. 2019;5. 10.1126/sciadv.aav1848. 10.1126/sciadv.aav1848 PMC6353624 30729162

[114] Sakaki K, Tashiro K, Kuhara S, Mihara K. Response of Genes Associated with Mitochondrial Function to Mild Heat Stress in Yeast *Saccharomyces cerevisiae*. J Biochem. 2003;134:373–84. 10.1093/jb/mvg155. 14561723 10.1093/jb/mvg155

[115] Varriale A. DNA Methylation, Epigenetics, and Evolution in Vertebrates: Facts and Challenges. Int J Evol Biol. 2014;2014:475981. 10.1155/2014/475981 24551476 10.1155/2014/475981 PMC3914449

[116] Mccaw BA, Stevenson TJ, Lancaster LT. Epigenetic responses to temperature and climate. Integr Comp Biol. 2020;60:1469–80. 10.1093/icb/icaa049. 32470117 10.1093/icb/icaa049

[117] Franks SJ, Hoffmann AA. Genetics of climate change adaptation. Annu Rev Genet. 2012;46:185–208.10.1146/annurev-genet-110711-155511 22934640 10.1146/annurev-genet-110711-155511

[118] Bräutigam K, Vining KJ, Lafon-Placette C, Fossdal CG, Mirouze M, Marcos JG, et al. Epigenetic regulation of adaptive responses of forest tree species to the environment. Ecol Evol. 2013;3:399–415. 10.1002/ece3.461. 23467802 10.1002/ece3.461 PMC3586649

[119] Sammarco I, Münzbergová Z, Latzel V. DNA methylation can mediate local adaptation and response to climate change in the clonal plant *Fragaria vesca*: evidence from a European-scale reciprocal transplant experiment. Front Plant Sci. 2022;13: 827166. 10.3389/fpls.2022.827166. 35295625 10.3389/fpls.2022.827166 PMC8919072

[120] Janzen DH. Why Mountain Passes are Higher in the Tropics. Am Nat. 1967;101:233–49.

[121] Pither J. Climate tolerance and interspecific variation in geographic range size. Proc R Soc Lond B Biol Sci. 2003;270:475–81. 10.1098/rspb.2002.2275. 10.1098/rspb.2002.2275 PMC1691271 12641901

[122] Jin Q, Chachar M, Ali A, Chachar Z, Zhang P, Riaz A, et al. Epigenetic Regulation for Heat Stress Adaptation in Plants: New Horizons for Crop Improvement under Climate Change. Agronomy. 2024;14:2105. 10.3390/agronomy14092105

[123] Fossdal CG, Krokene P, Olsen JE, Strimbeck R, Viejo M, Yakovlev I, et al. Epigenetic stress memory in gymnosperms. Plant Physiol. 2024;195:1117–33. 10.1093/plphys/kiae051. 38298164 10.1093/plphys/kiae051 PMC11142372

[124] Turchetti B, Marconi G, Sannino C, Buzzini P, Albertini E. DNA methylation changes induced by cold in psychrophilic and psychrotolerant *Naganishia* yeast species. Microorganisms. 2020;8: 296. 10.3390/microorganisms8020296. 32093408 10.3390/microorganisms8020296 PMC7074839

[125] Zhang W, Furtado K, Wu P, Zhou T, Chadwick R, Marzin C, et al. Increasing precipitation variability on daily-to-multiyear time scales in a warmer world. Sci Adv. 2021;7: eabf8021. 10.1126/sciadv.abf8021. 34321203 10.1126/sciadv.abf8021 PMC8318378

[126] Senckenberg Gesellschaft fuer Naturforschung. Umbilicaria spp. & Dermatocarpon miniatum. NCBI BioProject PRJNA281425. 2022. https://www.ncbi.nlm.nih.gov/bioproject/PRJNA820300/.

[127] Laetsch DR, Blaxter ML. Blobtools: interrogation of genome assemblies. F1000Res. 2017;6: 1287. 10.12688/f1000research.12232.1.

[128] Astashyn A, Tvedte ES, Sweeney D, Sapojnikov V, Bouk N, Joukov V, et al. Rapid and sensitive detection of genome contamination at scale with FCS-GX. Genome Biol. 2024;25:1–25. 10.1186/s13059-024-03198-7 38409096 10.1186/s13059-024-03198-7 PMC10898089

[129] Mendes FK, Vanderpool D, Fulton B, Hahn MW. Cafe 5 models variation in evolutionary rates among gene families. Bioinformatics. 2021;36:5516–8. 10.1093/bioinformatics/btaa1022. 33325502 10.1093/bioinformatics/btaa1022

[130] Emms DM, Kelly S. Orthofinder: phylogenetic orthology inference for comparative genomics. Genome Biol. 2019;20:1–14. 10.1186/s13059-019-1832-y. 31727128 10.1186/s13059-019-1832-y PMC6857279

[131] Emms DM, Kelly S. STAG: Species Tree Inference from All Genes. bioRxiv. 2018;267914. 10.1101/267914

[132] Williams L, Colesie C, Ullmann A, Westberg M, Wedin M, Büdel B. Lichen acclimation to changing environments: Photobiont switching vs. climate-specific uniqueness in *Psora decipiens*. Ecol Evol. 2017;7:2560–74. 10.1002/ece3.2809. 28428847 10.1002/ece3.2809 PMC5395455

[133] Yu G, Wang LG, Han Y, He QY. Clusterprofiler: an R package for comparing biological themes among gene clusters. OMICS. 2012;16:284–7. 10.1089/omi.2011.0118. 22455463 10.1089/omi.2011.0118 PMC3339379

[134] Supek F, Bošnjak M, Škunca N, Šmuc T. Revigo summarizes and visualizes long lists of gene ontology terms. PLoS One. 2011;6: e21800. 10.1371/journal.pone.0021800. 21789182 10.1371/journal.pone.0021800 PMC3138752

[135] Steenwyk JL, Buida TJ, Gonçalves C, Goltz DC, Morales G, Mead ME, et al. BioKIT: a versatile toolkit for processing and analyzing diverse types of sequence data. Genetics. 2022;221:iyac079. 10.1093/genetics/iyac079 35536198 10.1093/genetics/iyac079 PMC9252278

[136] R Core Team. R: A language and environment for statistical computing. R Foundation for Statistical Computing, Vienna, Austria. 2021. https://www.r-project.org/.

[137] Vacic V, Uversky VN, Dunker AK, Lonardi S. Composition profiler: a tool for discovery and visualization of amino acid composition differences. BMC Bioinformatics. 2007;8:1–7. 10.1186/1471-2105-8-211. 17578581 10.1186/1471-2105-8-211 PMC1914087

[138] Fick SE, Hijmans RJ. WorldClim 2: new 1-km spatial resolution climate surfaces for global land areas. Int J Climatol. 2017;37:4302–15.10.1002/joc.5086

[139] Körner C. The use of “altitude” in ecological research. Trends Ecol Evol. 2007;22:569–74. 10.1016/j.tree.2007.09.006 17988759 10.1016/j.tree.2007.09.006

[140] Broad Institute. Picard Toolkit. GitHub Repository. 2019. https://broadinstitute.github.io/picard/.

