# Supplementary material for: Temperature variability and other climatic attributes linked to genomic features in the lichen-forming fungal genus Umbilicaria
Source: BMC Biol. 2025 Oct 2;23:293. doi: 10.1186/s12915-025-02373-x (PMC12492740; doi:10.1186/s12915-025-02373-x)
Supplement: Supplementary file 2 — Addition File 2: Supplementary information and Figure S1-S15. Figure S1– Phylogram and chronogram. Figure S2 – Genome telomere ends. Figure S3 – Secondary metabolism gene trees. Figure S4-S5 – Amino acid differences among climate zones. Figure S6 – PCA statistics. Figure S7 – GC differences among climate zones. Figure S8 – Number of ENC in climate zones. Figure S9 – CAZymes in climate zones. Figure S10 – Correspondence analysis of RSCU. Figure S11 – BIOCLIM distribution in climate zones. Figure S12 – Methylation patterns. Figure S13 – Annotations of gene family changes in U. subpolyphylla. Figure S14 – Gene outliers in difference analyses. Figure S15 – ENC VS GC3. [file 12915_2025_2373_MOESM2_ESM.docx]

**Additional File 2**

**Supplementary Information and Figures**

Temperature variability and other climatic attributes linked to genomic features in the lichen-forming fungal genus *Umbilicaria*

Running title: Climate affects *Umbilicaria* genomes

Edgar L.Y. Wong^1-2^, Anjuli Calchera^1-2^, Jürgen Otte^1^, Imke Schmitt^1-2^

^1^ Senckenberg Biodiversity and Climate Research Centre, Frankfurt am Main, Germany

^2^ Goethe University Frankfurt, Department of Biosciences, Institute of Ecology, Evolution & Diversity, Frankfurt, Germany

Correspondence: [edgar.wong@senckenberg.de](mailto:edgar.wong@senckenberg.de)

1. **Results**

Gene gains in *U. subpolyphylla*

290 CAFE families in this study resulted in gene gain in all three *U. subpolyphylla* samples, whereas 12 CAFE families resulted in gene losses in all three samples. Among these gene gains*,* around half of the GO terms are associated with cellular catabolic processes regarding biological processes; major GO categories of molecular functions include zinc ion and DNA binding, ATP hydrolysis and hydrolase activity, catalytic activating, protein and enzyme binding (Additional File 1: Table S16; Additional File 2: Figure S14). InterPro families most annotated for in gene gains include those related to P-loop, Armadillo-type and WD40 structures (Additional File 1: Table S17). Among gene loss in *U. subpolyphylla,* regulation of cell cycle and proteolysis contributed to around half of the GO terms in biological processes, whereas hydrolase activating, protein binding, catalytic activity and transferase activities are the major categories for molecular functions (Additional File 1: Table S16; Additional File 2: Figure S14). The top annotated InterPro families for gene losses are associated with the alpha-beta hydrolase fold and zinc finger (Additional File 1: Table S17; Additional File 2: Figure S14). The COG for gene loss are mostly defence mechanisms (V), and posttranslational modification, protein turnover and chaperones (O) (Additional File 1: Table S18; Additional File 2: Figure S14). No GO terms were enriched (results not shown). Besides, *U. subpolyphylla* samples have more CAZymes in the following families than all *U. polyphylla* samples (Additional File 1: Table S19): AA1, CBM18, CE1, GH133, GH16, GH76, GT2_Chitin_synth, GT20, GT22, GT34, GT62, GT71. Among these, *U. subpolyphylla* has more annotated CAZymes in CE1 and all GT families than not only *U. polyphylla,* but all other studied samples.

Gene functions not climate-related in CAFE families, under significant selection, and with strong codon bias

***Clusters of orthologous groups (COGs) (Additional File 1: Table S18, Additional File 2: Figure S14a)***: In general, there are few annotations for categories: (N) cell motility, (V) defence mechanisms, (W) extracellular structures, and (Y) nuclear structure. χ^2^ test of independence showed that there is significant association between gene group and COG (χ^2^ = 485.81, df = 161, p-value < 2.2x10^-16^). All climate zones selection groups, except negatively selected genes in the Mediterranean zone, show a positive association with number of annotations in (G) carbohydrate transport and metabolism. Genes under negative selection in all three zones showed positive correlation with annotations in (D) cell cycle control, cell division and chromosome partitioning. Single copy orthologues with strong codon bias (ENC ≤ 35) have particularly positive correlation with annotations in (H) coenzyme transport and metabolism, whereas all orthologues with strong codon bias showed strong negative association with annotations in (D) cell cycle control, cell division, chromosome partitioning, (G) carbohydrate transport and mechanism, and (T) signal transduction mechanisms.

Fewer than 40 genes under significant positive selection had a COG annotation, hence the results are not shown in Additional File 2: Figure S14a (full results in Additional File 1: Table S18). For the limited number of genes annotated, all three climate zones had genes annotated for (G) carbohydrate transport and metabolism and (Z) cytoskeleton. The alpine and cold temperate zone both had annotations for (T) signal transduction mechanisms; whereas the cold temperate and Mediterranean zone both had annotations for (C) energy production and conversion and (L) replication, recombination and repair.

***InterPro annotations (Additional File 1: Table S20-26, Additional File 2: Figure S14c)****:* To get an overview of protein domain and superfamily annotations, we compared 20 annotations with the most counts in each of the eight gene group [CAFE family genes, genes under significant positive and negative selection in each climate zone, genes with strong codon bias (ENC ≤ 35)]. Ankyrin repeat (IPR002110, IPR036770) is found in the top hits of all eight groups. P-loop containing nucleoside triphosphate hydrolase (P-loop NTPases) (OPR027417) was found in all groups except for positively selected genes of the alpine zone. Acyl-CoA N-acyltransferase (IPR016181) and Fungal-type protein kinase (IPR040976) are annotated for positively selected genes from all climate zones. On the contrary, helicase (IPR014001, IPR001650) is annotated for negatively selected genes from all climate zones. Armadillo-type fold (IPR016024), cytochrome P450 (IPR001128, IPR036396), major facilitator superfamily domain (IPR020846), MFS transporter superfamily (IPR036259), and NAD(P)-binding domain superfamily (IPR036291) are annotated for negatively selected genes from all climate zones and CAFE family genes; with the last one also annotated for genes with strong codon bias. Protein kinase (IPR000719, IPR011009) is annotated for all groups except genes with strong codon bias. Other annotations that were present in the top hits in at least five gene groups include tetratricopeptide (IPR019734, IPR011990), WD40 repeats (IPR001680, IPR015943), and alpha/Beta hydrolase fold (IPR029058).

Genes with strong codon bias

Comparing the relationship between ENC and GC3 (Additional File 2: Figure S15), single-copy orthologues in all species scattered around the expected curve; for all orthologues, the majority of genes in all species appear below the expected curve. Focusing on genes with a strong codon bias (ENC ≤ 35), single-copy genes that had a strong codon bias in 10 or more (> 33.3%) samples include farnesyl pyrophosphate synthetase (20 samples), phospholipid metabolism protein (13 samples), and dephospho-CoA kinase cab5 (12 samples) (Figure 13b, Additional File 1: Table S27). For all other orthologues (that has other copies), the genes that fit the same criteria include F1F0 ATP synthase subunit e, mitochondrial (16 samples), trafficking protein particle complex subunit 31 (16 samples), oxidation resistance protein 1 (13 samples), protein csh3 (11 samples), thioredoxin trx1 (11 samples), and superoxide dismutase [Cu-Zn] (10 samples) (Additional File 1: Table S4, Additional File 2: Figure S14b). No gene has a strong codon bias in all 27 samples, or in all samples within the same climate zone (Additional File 1: Table S27).

Phylogenetic signal in correlation between bioclimatic variables and genome features

Pagel’s λ ranged from 0.27 to 0.92 (Additional File 1: Table S28) for the six correlations (Figure 4), indicating variable levels of phylogenetic signals in the correlations. However, estimated errors are also high, with at least 0.41; and as high as 0.97 in the 95% confidence range (CI) (upper CI – lower CI) (Additional File 1: Table S28). Together with the fact that Pagel’s λ estimations produce high type II errors for small phylogenies (<20 species) [1], we conclude that the sample size in this study does not provide enough power to confidently estimate phylogenetic signal and should be disregarded.

1. **Discussion**

Taxonomic relationship between *U. subpolyphylla and U. polyphylla*

A previous study showed that *U. subpolyphylla* formed a sub-clade within *U. polyphylla* in the ITS phylogeny, whereas the two species formed distinct clades in the mitochondrial LSU phylogeny and combined ITS-mtLSU phylogeny [2]. In our phylogeny based on all orthologous genes (Figure 1a, Additional File 2: Figure S1), the seven samples of *U. subpolyphylla* and *U. polyphylla* were all collected on the island of Corsica, France at different elevations. They form a monophyletic clade together, but the two species are intermixed within this clade. Thus, we consider *U. subpolyphylla* and *U. polyphylla* a phylogenetically unresolvable species complex. Interestingly, in addition to the morphological differences reported by Davydov *et al.*, (2019), we found consistent genomic patterns that distinguish the two taxa, including *U. polyphylla* having more contracted gene families, *U. subpolyphylla* having more expanded gene families, more annotated genes (with gene gains from 290 CAFE families), bigger CAZyme families, lower codon bias (higher ENC) (Figure 1a). The two taxa also have different RSCU patterns (Additional File 2: Figure S10). *U. subpolyphylla* also has more annotated CAZymes in all glycosyltransferase (GT) families than any other species in this study. It is unlikely that these differences are environmentally driven, as both species occur in both the Mediterranean and cold temperate climate zones. Future research is required to study the underlying mechanisms that drive these differences. Interestingly, certain GO categories are shared by both gained and lost genes in *U. subpolyphylla,* such as hydrolase activity and protein binding. Zinc ion binding is a major GO category among gene gains, while zinc finger was among top annotated InterPro families for gene losses. These could suggest that the functions of genes lost in *U. subpolyphylla* are still essential to their lifestyle, yet they ‘switch’ to new genes to perform those functions.

Genes and functions universally important for lichenised fungi in all climate zones

Most gene groups under positive or negative selection showed positive association with annotations in carbohydrate transport and metabolism (G), which could be related to nutrient transport between lichen symbionts and responses to climate (e.g. in terms of maintaining membrane fluidity). A few gene families are present as outliers in multiple analyses based on InterPro annotations (Additional File 2: Figure S14c, Additional File 1: Table S20-25). Gene families related to ankyrin repeats are present in the top counts in all eight analyses and genes related to tetratricopeptide are present in five analyses, indicating that they are universally important in all samples. They have undergone significant changes in gene family size, with different genes under both positive and negative selection and have strong codon bias. They are also abundant in eukaryotic genomes. For instance, ankyrin repeat domains are found in around 6% of eukaryotic protein sequences [3]. Both types of repeats are involved in protein-protein interactions and have been reported to have a role in symbiosis (and host-pathogen interactions). This includes symbiosis in arbuscular mycorrhizal fungi [4], as well as between bacteria and marine sponges, amoebae or insects [5–9]. Specifically, these repeats were hypothesised to be involved in symbiotic (or host) interactions [6, 8, 9], and evasion of host immune response [6].Another superfamily of genes related to MFS transporters is present in the top counts among negatively selected genes for all climate zones, again suggesting their important role in membrane transport of the lichen symbiosis. Although this superfamily was found to significantly contract in lichen-forming fungi, they were still found to be crucial at different stages of lichenisation [10]. Apart from lichens, MFS transporters’ functions in other types of symbioses are well-documented as well, including that between legume and rhizobium [11], bacteria and plants or animals [12], aphids and bacteria [13], cnidarian and dinoflagellate [14], and in arbuscular mycorrhiza [15]. Gene families related to WD40 is also of interest, as it has been associated with abiotic stress tolerance in plants, including drought and high temperature stress [16–19].

Methylation patterns among different climate zones within species

Within the same species, although methylation seems to be higher in the samples from the colder climate, this is not the case for all genomic windows studied. Certain genomic windows could harbour many genes important for responses to higher temperatures or environmental stressors specific to the warmer climate zones, leading to methylation being lower in these regions in samples from the colder climate zone. It could also be caused by merely natural variation in methylation rates across genomes or incomplete mapping of methylated sites to the *U. pustulata* reference genome. The latter could also explain the huge variation in methylation rate among species as well, which is extremely low compared to 0.08-12.8% in other Ascomycota species with 5mC data available (reviewed in Nai *et al.*, 2021). The higher methylation rate in samples from the colder climate zone was only significant in four out of seven species. Same as above, potential incomplete mapping of methylation sites to the *U. pustulata* reference genome, and hence variable numbers of methylated sites analysed for each genomic window, could introduce biases in these results. Future research including generation of more complete reference genomes for other species and methylation specific sequencing techniques (such as bisulfite sequencing) are required to further explore this subject.

1. **Methods**

A total of 27 samples were collected and used, 15 of which have been included in previous publications (Table 1, Additional File 1: Table S1). Whole lichen thalli (dry) were collected from the inhabited rock surfaces and stored in sealed plastic bags until sample processing. Prior to DNA extraction, lichen thalli were first rinsed with distilled water to remove any contaminants. Lichen thalli were then grounded into fine powder in liquid nitrogen. Genomic DNA was extracted using a modified CTAB method following [21]. Purified DNA was sent to either the Max Planck Institute of Molecular Cell Biology and Genetic (Dresden, Germany) (for *U. pustulata*) or Radboud University Medical Center (Nijmegen, Netherlands) (all other samples) to prepare PacBio sequencing libraries. Resulting libraries were sequenced using either the CLR or CCS sequencing mode (Additional File 1: Table S1) on one SMRT cell each, with adaptors removed by the sequencing facility subsequently.

Sampling for RNA sequencing

We also sequenced RNA of seven out of 11 studied species (10 samples in total) to aid in genome annotation. Samples used here come from a different individual of the same species. Lichen thalli were pulverised with liquid nitrogen. RNA was then isolated following either [22] for samples with abundant input material, and [23] for samples with low input material. Purified RNA was sent to Novogene for paired-end 150bp sequencing on the Illumina NovaSeq platform, with an output of 5 - 7Gb per sample. Removal of adaptors and low-quality reads were performed by Novogene.

Genome assembly, filtering and annotation

We either re-filtered or re-assembled the 15 previously reported genomes because (1) updated versions of programmes for genome assembly and contamination filtering are available; (2) a standard analytical protocol for all genomes ensures directly comparable genomes (Table 1, Additional File 1: Table S1).

The previously published genome of *U. pustulata* (TBG2345) (GenBank assembly no: ASM2318457v1) was re-filtered to serve as the reference genome. The genome was first blasted (v2.15.0) against the NCBI *nt* database as of April, 2022, using default settings. A later version of the *nt* database was not used as genomes that had been re-assembled or re-filtered in this studied were published after that date and the use of a later database would result in self matches, thus masking potential contaminations previously undetected. The blast output was run through the *BlobTools* v1.1.1 pipeline [24]. Scaffolds (regardless of length) with GC content outside of 40 - 60% and the contig’s top hit not assigned to *Ascomycota* were removed. The range of 40 - 60% was chosen as this is the range where Lecanoromycetes (the class that *Umbilicaria* belongs to) genomes lie [25]. *FCS-adapter* and *FCS-GX* v0.5.0 with database gx-version build 2023-01-24 (git:v0.3.0-151-g9aad15db) [26] was subsequently run with the filtered assembly to detect any further contamination. Contaminated whole scaffolds or contaminated sequences within scaffolds were removed following *FCS*’s detection. BUSCO v.5.6.1 [27] was run using the *fungi_odb10* database, before and after running *FCS* to ensure that removal of identified contamination did not result in drastic decrease in the percentage of BUSCO genes.

All newly assembled genomes were sequenced using the CCS mode. For samples in which a reads file was supplied, HiFi reads were extracted using the *extracthifi* option, followed by indexing using the *pbindex* option from the *pbtk* package v3.1.0 (PacificBiosciences). For samples in which a subreads file was supplied, the *pbccs* package v6.4.0 (PacificBiosciences) was used to extract consensus reads with the *--hifi-kinetics* flag. All resulting bam files were converted into the fasta format using the *bam2fasta* option in the *pbtk* package. Metagenomes of whole lichen communities were assembled using *flye* v2.9.1 [28], with the flags *--meta* and *--pacbio-hifi*.

All newly assembled, re-assembled and previously published metagenomes were filtered similar to the procedures above for preparing the reference genome. They were blasted against the decontaminated *U. pustulata* (TBG2345) genome, in addition to the NCBI *nt* database (as of April 2022), using default setting. In the *Blobtools* v.1.1.1 pipeline, all contigs (regardless of length) with GC content outside of 40 - 60% and the contig’s top hit not matched to *U. pustulata* (TBG2345) were removed. The *clean* command (with *--exhaustive* flag) in the *funannotate* pipeline v1.8.15 [29] was used to remove duplicated contigs. Afterwards, *FCS* and BUSCO were run to further remove contaminations, in the same way as described above. For final genome completeness scores, we ran *BUSCO* v.5.8.2 with the lineage dataset ascomycota_odb12 (2025-04-11) with *augustus* as gene finder in optimized self-training mode. Chromosome completeness of all genome assemblies was examined using *Tapestry* v1.0.1 [30], in which the repeat sequence “TTAGGG”/”CCCTAA” was used to detect telomere ends typical of chromosomal ends in eukaryotes.

All genomes were annotated using the rest of the *funannotate* pipeline [29]. The *sort* and *mask* commands were used to sort contigs and soft-mask assemblies respectively. For samples in which RNA sequences of the same species were available (Additional File 1: Table S1), the *train* command was used for genome-guided transcriptome assemblies (*Trinity* v2.8.5) and spliced alignment assemblies (*PASA* v2.5.2) to train gene predictions in the next step. These samples were then run through the *predict* command for gene prediction, using a combination of tools such as *AUGUSTUS* 3.5.0 [31] and *GeneMark* [32] (details Palmer and Stajich, 2019). This is followed by the *update* command, which updates predicted gene models with the RNA data. For samples without RNA sequences, the *predict* command was run (but not *train* and *update*) with extra flags *--protein_evidence* and *--protein_alignments*, using the Trinity assembly and predicted proteins of *U. pustulata* (TBG2345). Although potential biases could be introduced by using a different species, using a closely-related species’ transcriptome assembly would still be better than using the default prediction without any reference. Also, RNA extracted from the basal taxon in the phylogeny presented, *U. pustulata*, was available. Hence the gene models in this reference species have been validated with RNA evidence, yielding high-quality protein models that can be used to direct the ab initio gene prediction algorithms for other species without conspecific RNA data. For all samples, outputs of the *predict* commands were checked for problematic gene models and duplicated coding sequences, which were corrected manually in the annotation files. Corrected annotation files were subsequently used in running the *fix* command to generate an updated set of predictions. Using these updated predictions, *InterproScan5* v5.61-93.0 [33] was run locally using the *iprscan* command; *signalP* v6.0 [34] and *antismash* v7.1.0 [35] were also run separately (not part of the *funannotate* pipeline). Using the *InterproScan*, *signalP* and *antismash* results, the *annotate* command was used to functionally annotates all gene predictions using PFAM v35.0, InterPro v5.61-93.0, EggNog (EggNog Mapper v2.1.10, DB v5.0.2), UniProtKB v2023_02, MEROPS v12.0, CAZyme (dbCAN v11.0), and GO ontology v2023-04-01 (details in [29]).

Protein domains, COG and GO enrichment in target genes

Potential functions of genes with significance in various analyses (gene family expansion and contraction, positive and negatives selection (dN/dS), ENC ≤ 35) were examined using annotations carried out with the *funannotate* pipeline as described above. First, gene names of all targets were summarised for each analysis. Any common genes were compared among genes under positively and negatively selection in each climate zone. Secondly, InterPro protein superfamilies and protein domains were extracted and summarised for each target group. Third, cluster of orthologues (COG) were summarised into the 24 categories (see Additional File 2: Figure S14) and compared among the target groups through percentage of all COGs. GO enrichment was not tested for genes with strong codon bias for each genome. This is because it is of interest to test whether certain genes are conserved across samples, compared to conserved functions that could be encoded by different genes (which would suggest divergent evolution). Hence, we compared the genes annotated for each sample and identified ones that repeatedly showed strong codon bias in multiple samples.

Assessing phylogenetic signal in correlations

Phylogenetic signal in these correlations were assessed through Pagel’s λ under the Bayesian generalised linear multivariate multilevel model, using the *R* (v4.2.1) package *brms* v2.22.0 [36]. Pagel’s λ typically ranges between 0 and 1, where λ = 0 signifies the lack of phylogenetic signal (high evolutionary rate and little effect of species relatedness in correlation) and λ = 1 signifies strong phylogenetic signal. It is a rate-independent measure of the degree to which closely-related species resemble one-another relative to a Brownian motion expectation. In R, the phylogeny with species names as node tips (instead of sample identifiers) was used. Species means and deviation from species means for each sample were estimated for PC values (representing collinear bioclimatic variables). Prior values for running the Bayesian multilevel model were obtained using the function *get_prior*, which was then used in running the *brm* function. The *adapt_delta* option was increased if there was any divergent transition after warmup in the analysis until no transitions were detected, following the package’s recommendation. Pagel’s λ was then estimated using the *hypothesis* function.

1. **References**

1. Münkemüller T, Lavergne S, Bzeznik B, Dray S, Jombart T, Schiffers K, et al. How to measure and test phylogenetic signal. Methods Ecol Evol. 2012;3:743–56. DOI: 10.1111/j.2041-210X.2012.00196.x

2. Davydov EA, Peršoh D, Rambold G. Umbilicariaceae (lichenized Ascomycota) – Trait evolution and a new generic concept. Taxon. 2019;66:1282–303. DOI: 10.12705/666.2

3. Barrick D, Ferreiro DU, Komives EA. Folding landscapes of ankyrin repeat proteins: experiments meet theory. Curr Opin Struct Biol. 2008;18:27–34. DOI: 10.1016/j.sbi.2007.12.004

4. Lindsay PL, Ivanov S, Pumplin N, Zhang X, Harrison MJ. Distinct ankyrin repeat subdomains control VAPYRIN locations and intracellular accommodation functions during arbuscular mycorrhizal symbiosis. Nat Commun. 2022;13:1–15. DOI: 10.1038/s41467-022-32124-3

5. Iturbe-Ormaetxe I, Burke GR, Riegler M, O’Neill SL. Distribution, expression, and motif variability of ankyrin domain genes in *Wolbachia pipientis*. J Bacteriol. 2005;187:5136–45. DOI: 10.1128/JB.187.15.5136-5145.2005

6. Alex A, Antunes A. Whole Genome Sequencing of the Symbiont *Pseudovibrio* sp. from the Intertidal Marine Sponge *Polymastia penicillus* Revealed a Gene Repertoire for Host-Switching Permissive Lifestyle. Genome Biol Evol. 2015;7:3022–32. DOI: 10.1093/gbe/evv199

7. Thomas T, Rusch D, DeMaere MZ, Yung PY, Lewis M, Halpern A, et al. Functional genomic signatures of sponge bacteria reveal unique and shared features of symbiosis. ISME J. 2010;4:1557–67. DOI: 10.1038/ismej.2010.74

8. Mann E, Stouthamer CM, Kelly SE, Dzieciol M, Hunter MS, Schmitz-Esser S. Transcriptome Sequencing Reveals Novel Candidate Genes for *Cardinium hertigii* -Caused Cytoplasmic Incompatibility and Host-Cell Interaction. mSystems. 2017;2:e00141-17. DOI: 10.1128/mSystems.00141-17

9. Schulz F, Martijn J, Wascher F, Lagkouvardos I, Kostanjšek R, Ettema TJG, et al. A Rickettsiales symbiont of amoebae with ancient features. Environ Microbiol. 2016;18:2326–42. DOI: 10.1111/1462-2920.12881

10. Song H, Kim KT, Park SY, Lee GW, Choi J, Jeon J, et al. A comparative genomic analysis of lichen-forming fungi reveals new insights into fungal lifestyles. Sci Rep. 2022;12:1–14. DOI: 10.1038/s41598-022-14340-5

11. Vincill ED, Szczyglowski K, Roberts DM. GmN70 and LjN70. Anion Transporters of the Symbiosome Membrane of Nodules with a Transport Preference for Nitrate. Plant Physiol. 2005;137:1435–44. DOI: 10.1104/pp.104.051953

12. Pasqua M, Grossi M, Zennaro A, Fanelli G, Micheli G, Barras F, et al. The Varied Role of Efflux Pumps of the MFS Family in the Interplay of Bacteria with Animal and Plant Cells. Microorg. 2019;7:285. DOI: 10.3390/microorganisms7090285

13. Charles H, Balmand S, Lamelas A, Cottret L, Pérez-Brocal V, Burdin B, et al. A Genomic Reappraisal of Symbiotic Function in the Aphid/*Buchnera* Symbiosis: Reduced Transporter Sets and Variable Membrane Organisations. PLoS One. 2011;6:e29096. DOI: 10.1371/journal.pone.0029096

14. Sproles AE, Kirk NL, Kitchen SA, Oakley CA, Grossman AR, Weis VM, et al. Phylogenetic characterization of transporter proteins in the cnidarian-dinoflagellate symbiosis. Mol Phylogenet Evol. 2018;120:307–20. DOI: 10.1016/j.ympev.2017.12.007

15. Zhang M, Zhong X, Li M, Yang X, Abou Elwafa SF, Albaqami M, et al. Genome-wide analyses of the Nodulin-like gene family in bread wheat revealed its potential roles during arbuscular mycorrhizal symbiosis. Int J Biol Macromol. 2022;201:424–36. DOI: 10.1016/j.ijbiomac.2022.01.076

16. Kong D, Li M, Dong Z, Ji H, Li X. Identification of TaWD40D, a wheat WD40 repeat-containing protein that is associated with plant tolerance to abiotic stresses. Plant Cell Rep. 2015;34:395–410. DOI: 10.1007/s00299-014-1717-1

17. Çelik H, Aravena A, Turgut Kara N. Bioinformatics and gene expression analysis of the legume F-box/WD40 proteins in NaCl and high temperature stress. Genet Resour Crop Evol. 2023;70:2637–55. DOI: 10.1007/s10722-023-01592-x

18. Lee S, Lee J, Paek KH, Kwon SY, Cho HS, Kim SJ, et al. A novel WD40 protein, BnSWD1, is involved in salt stress in *Brassica napus*. Plant Biotechnol Rep. 2010;4:165–72. DOI: 10.1007/s11816-010-0131-6

19. Liu WC, Li YH, Yuan HM, Zhang BL, Zhai S, Lu YT. WD40-REPEAT 5a functions in drought stress tolerance by regulating nitric oxide accumulation in *Arabidopsis*. Plant Cell Environ. 2017;40:543–52. DOI: 10.1111/pce.12723

20. Nai YS, Huang YC, Yen MR, Chen PY. Diversity of Fungal DNA Methyltransferases and Their Association With DNA Methylation Patterns. Front Microbiol. 2021;11:616922. DOI: 10.3389/fmicb.2020.616922

21. Merges D, Dal Grande F, Greve C, Otte J, Schmitt I. Virus diversity in metagenomes of a lichen symbiosis (*Umbilicaria phaea*): complete viral genomes, putative hosts and elevational distributions. Environ Microbiol. 2021;23:6637–50. DOI: 10.1111/1462-2920.15802

22. Rubio-Piña JA, Zapata-Pérez O. Isolation of total RNA from tissues rich in polyphenols and polysaccharides of mangrove plants. Electron J Biotechnol. 2011;14:11. DOI: 10.2225/vol14-issue5-fulltext-8

23. Ahmad N, Ritz M, Calchera A, Otte J, Schmitt I, Brueck T, et al. Biosynthetic Potential of Hypogymnia Holobionts: Insights into Secondary Metabolite Pathways. J Fungi. 2023;9:546. DOI: 10.3390/jof9050546

24. Laetsch DR, Blaxter ML. BlobTools: Interrogation of genome assemblies [version 1; peer review: 2 approved with reservations]. F1000Research 2017. 2017;6:1287. DOI: 10.12688/f1000research.12232.1

25. Resl P, Bujold AR, Tagirdzhanova G, Meidl P, Freire Rallo S, Kono M, et al. Large differences in carbohydrate degradation and transport potential among lichen fungal symbionts. Nat Commun. 2022;13:1–13. DOI: 10.1038/s41467-022-30218-6

26. Astashyn A, Tvedte ES, Sweeney D, Sapojnikov V, Bouk N, Joukov V, et al. Rapid and sensitive detection of genome contamination at scale with FCS-GX. Genome Biol. 2024;25:1–25. DOI: 10.1186/s13059-024-03198-7

27. Manni M, Berkeley MR, Seppey M, Zdobnov EM. BUSCO: Assessing Genomic Data Quality and Beyond. Curr Protoc. 2021;1:e323. DOI: 10.1002/cpz1.323

28. Kolmogorov M, Bickhart DM, Behsaz B, Gurevich A, Rayko M, Shin SB, et al. metaFlye: scalable long-read metagenome assembly using repeat graphs. Nat Methods. 2020;17:1103–10. DOI: 10.1038/s41592-020-00971-x

29. Palmer J, Stajich J. nextgenusfs/funannotate: funannotate v1.5.3. Zenodo. 2019. DOI: 10.5281/zenodo.2604804.

30. Davey JW, Davis SJ, Mottram JC, Ashton PD. Tapestry: validate and edit small eukaryotic genome assemblies with long reads. bioRxiv. 2020. DOI: 10.1101/2020.04.24.059402.

31. Stanke M, Tzvetkova A, Morgenstern B. AUGUSTUS at EGASP: using EST, protein and genomic alignments for improved gene prediction in the human genome. Genome Biol . 2006;7:1–8. DOI: 10.1186/gb-2006-7-s1-s11

32. Ter-Hovhannisyan V, Lomsadze A, Chernoff YO, Borodovsky M. Gene prediction in novel fungal genomes using an ab initio algorithm with unsupervised training. Genome Res. 2008;18:1979–90. DOI: 10.1101/gr.081612.108

33. Jones P, Binns D, Chang HY, Fraser M, Li W, McAnulla C, et al. InterProScan 5: genome-scale protein function classification. Bioinformatics. 2014;30:1236–40. DOI: 10.1093/bioinformatics/btu031

34. Teufel F, Almagro Armenteros JJ, Johansen AR, Gíslason MH, Pihl SI, Tsirigos KD, et al. SignalP 6.0 predicts all five types of signal peptides using protein language models. Nat Biotechnol. 2022;40:1023–5. DOI: 10.1038/s41587-021-01156-3

35. Blin K, Shaw S, Kloosterman AM, Charlop-Powers Z, Van Wezel GP, Medema MH, et al. antiSMASH 6.0: improving cluster detection and comparison capabilities. Nucleic Acids Res. 2021;49:W29–35. DOI: 10.1093/nar/gkab335

36. Bürkner PC. brms: An R Package for Bayesian Multilevel Models Using Stan. J Stat Softw. 2017;80:1–28. DOI: 10.18637/jss.v080.i01

37. Williams L, Colesie C, Ullmann A, Westberg M, Wedin M, Büdel B. Lichen acclimation to changing environments: Photobiont switching vs. climate-specific uniqueness in *Psora* *decipiens*. Ecol Evol. 2017;7:2560–74. DOI: 10.1002/ece3.2809

Supplementary Figures


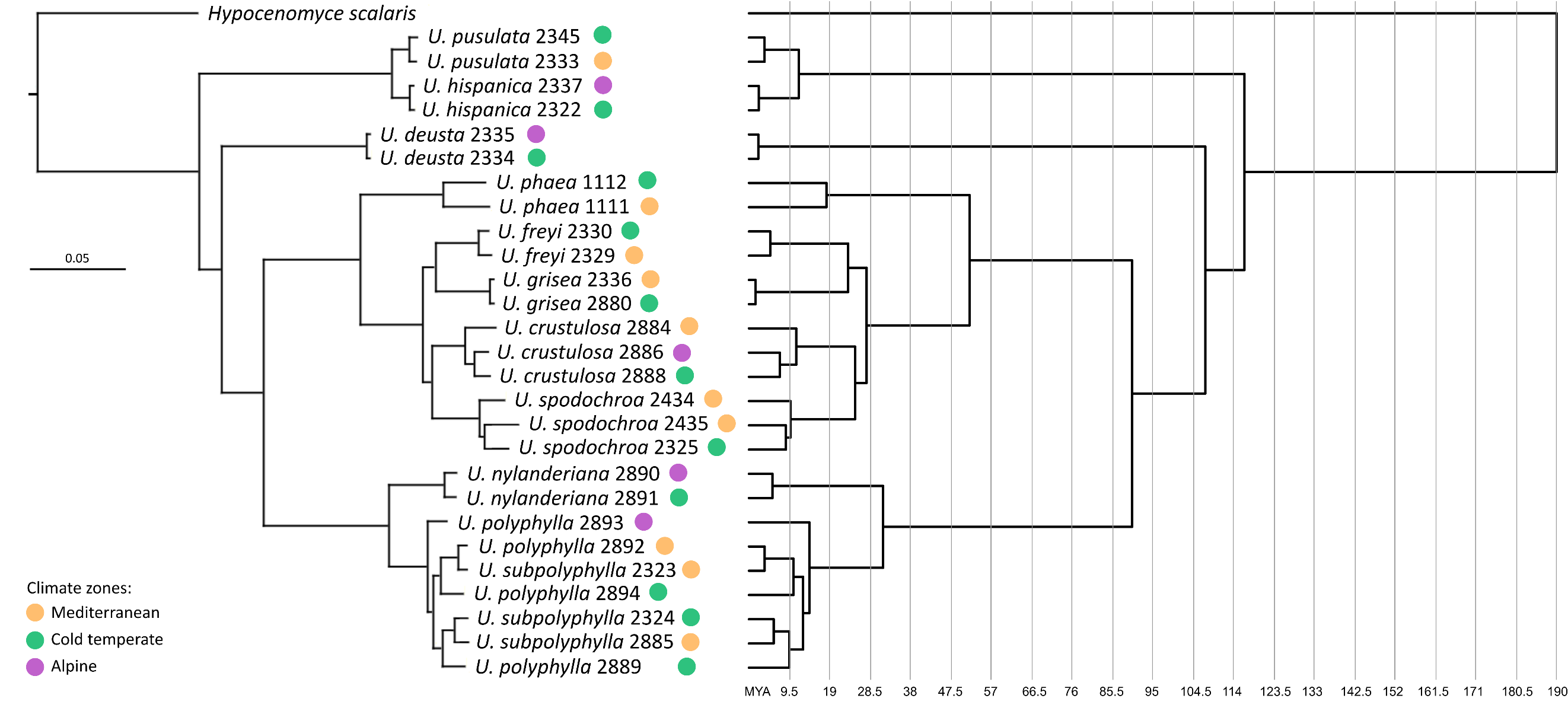


**Figure S1.** Phylogeny generated using concatenated alignments of all orthologous groups identified by OrthoFinder. Colours of circles denote the climate zone where the sample was collected. Left: phylogram based on molecular distance. Right: chronogram scaled using a root age of 190 million years ago [37].

**
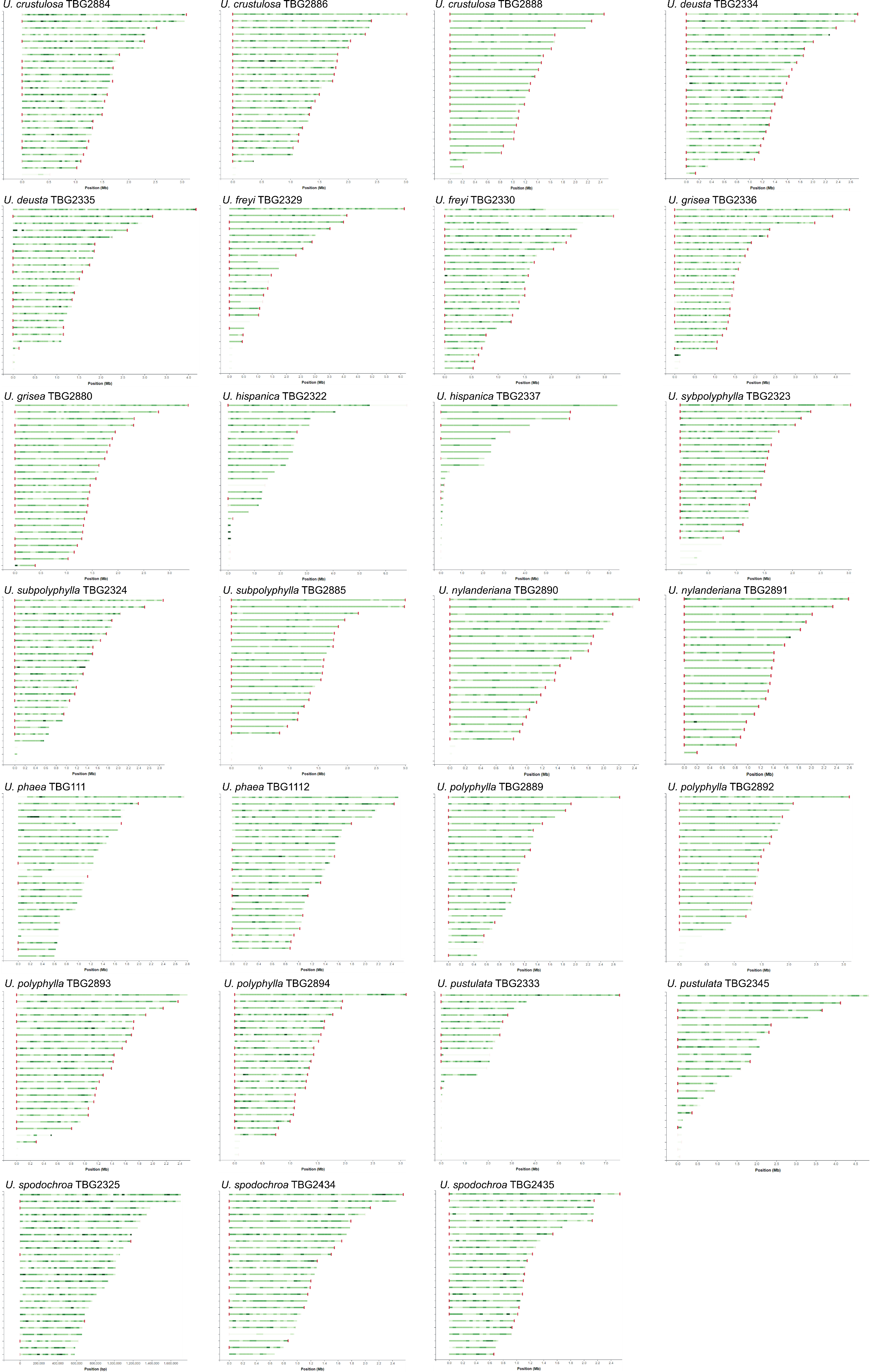
 Figure S2**. Figures showing telomere ends detected in each assembled contig or scaffold by *tapestry*. If the assembled genome has more than 25 scaffolds or contigs, only the first 25 are shown.

**
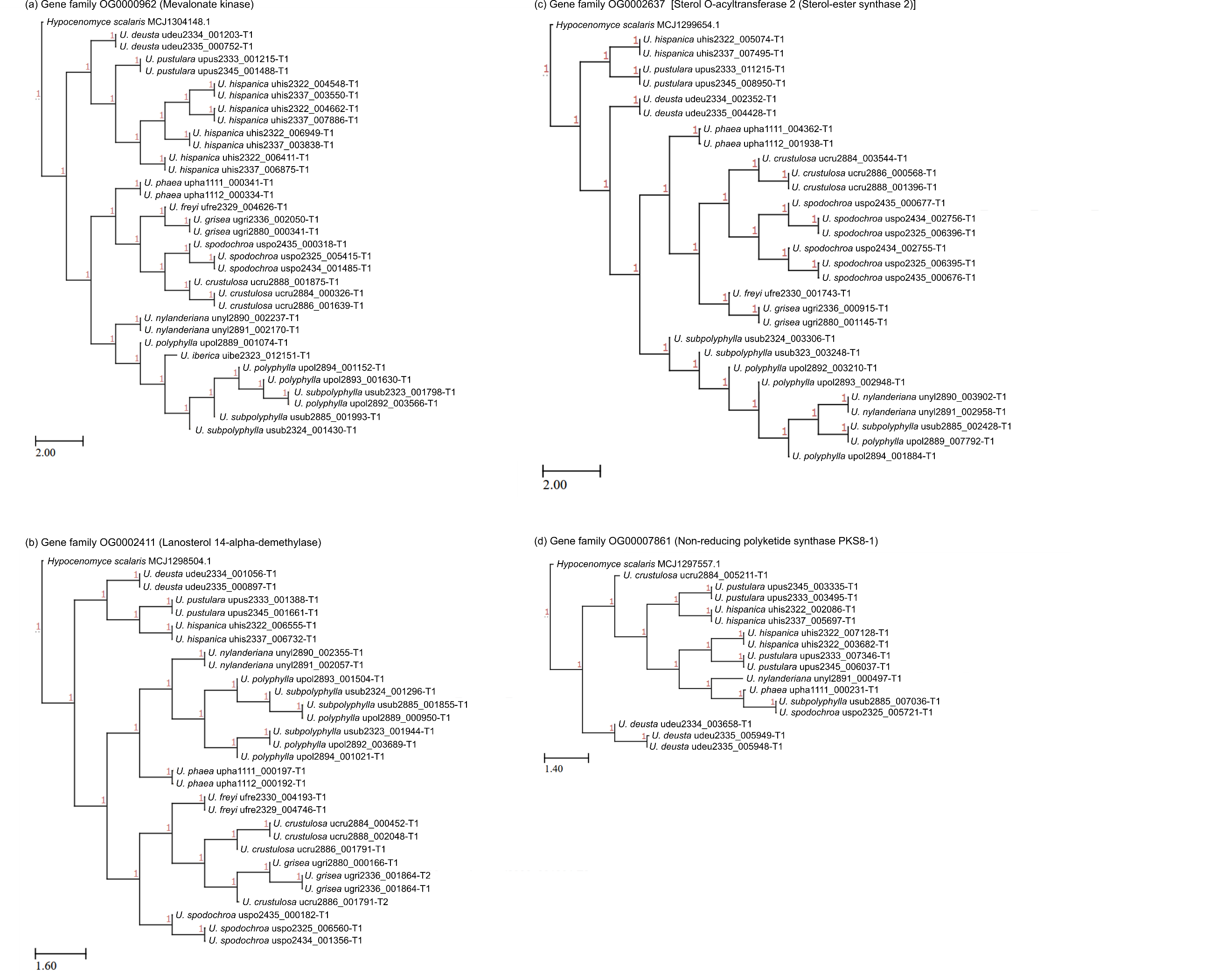
 Figure S3.** Gene trees of the four orthologous groups with annotated genes associated with secondary metabolism (output from OrthoFinder).


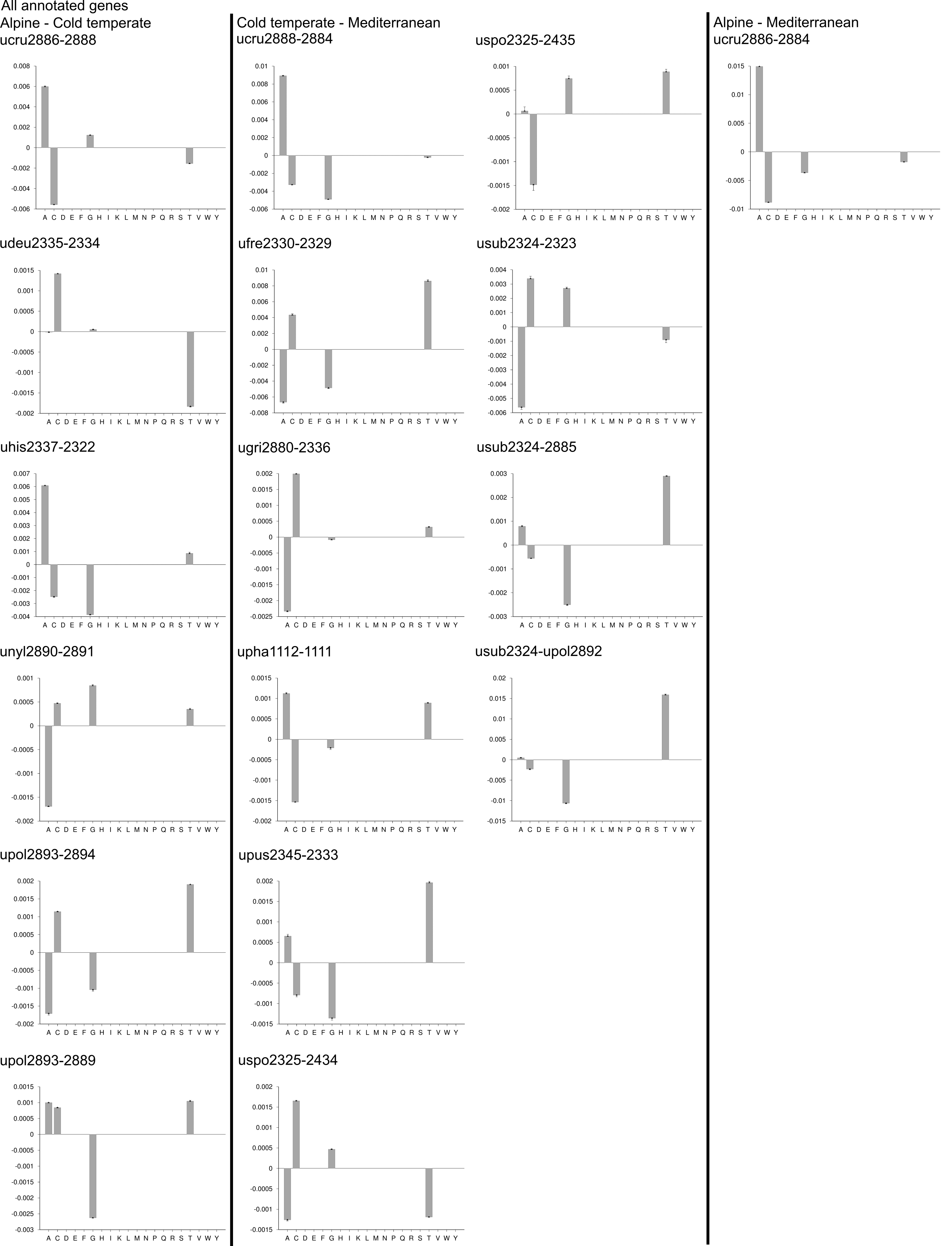
 **Figure S4.** Significant differences in amino acids encoded by all annotated genes between samples collected from different climate zones within each species. Letters on the x-axis denote standard abbreviations for amino acids.

**
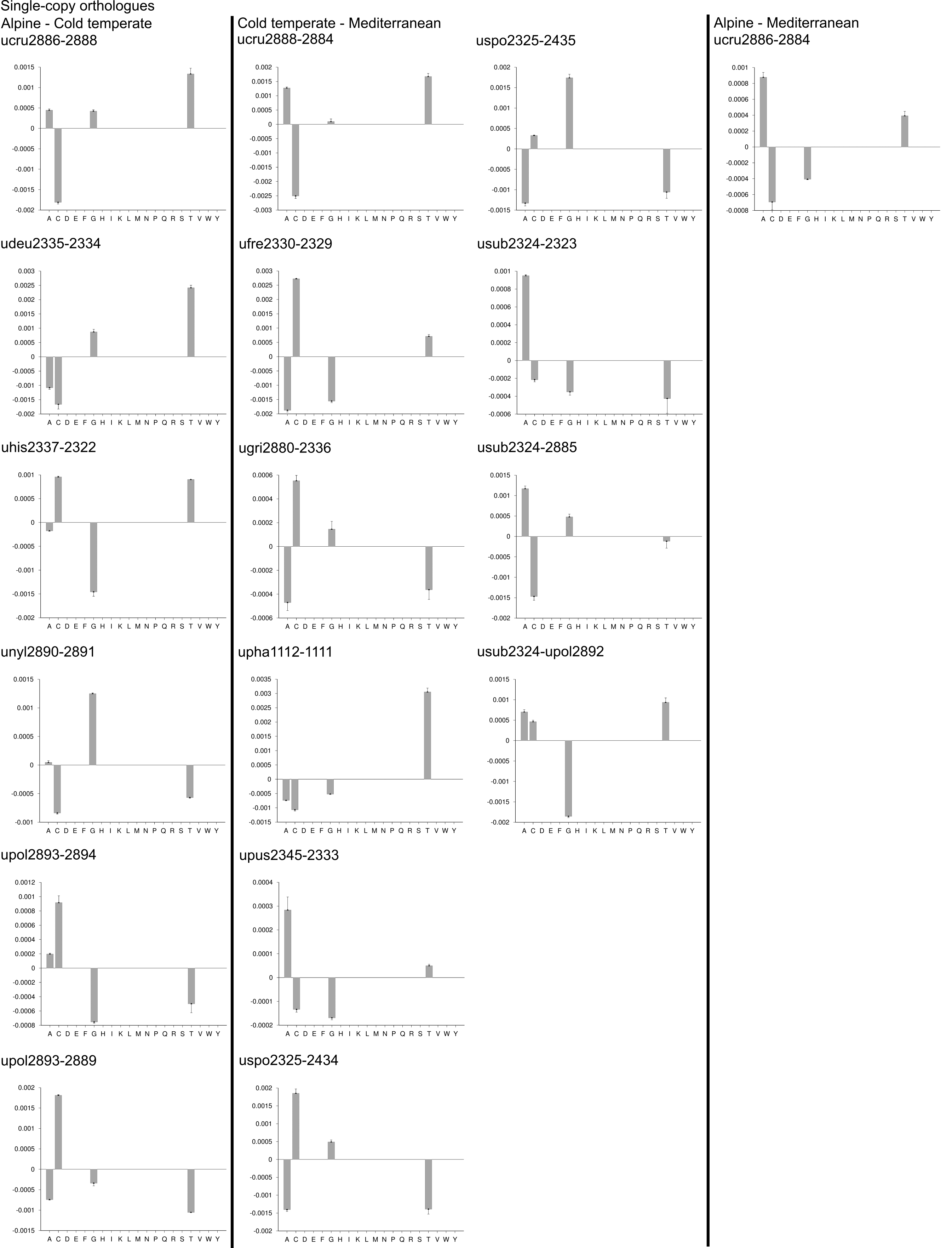
 Figure S5.** Significant differences in amino acids encoded by single-copy orthologues between samples collected from different climate zones within each species. Letters on the x-axis denote standard abbreviations for amino acids.

**
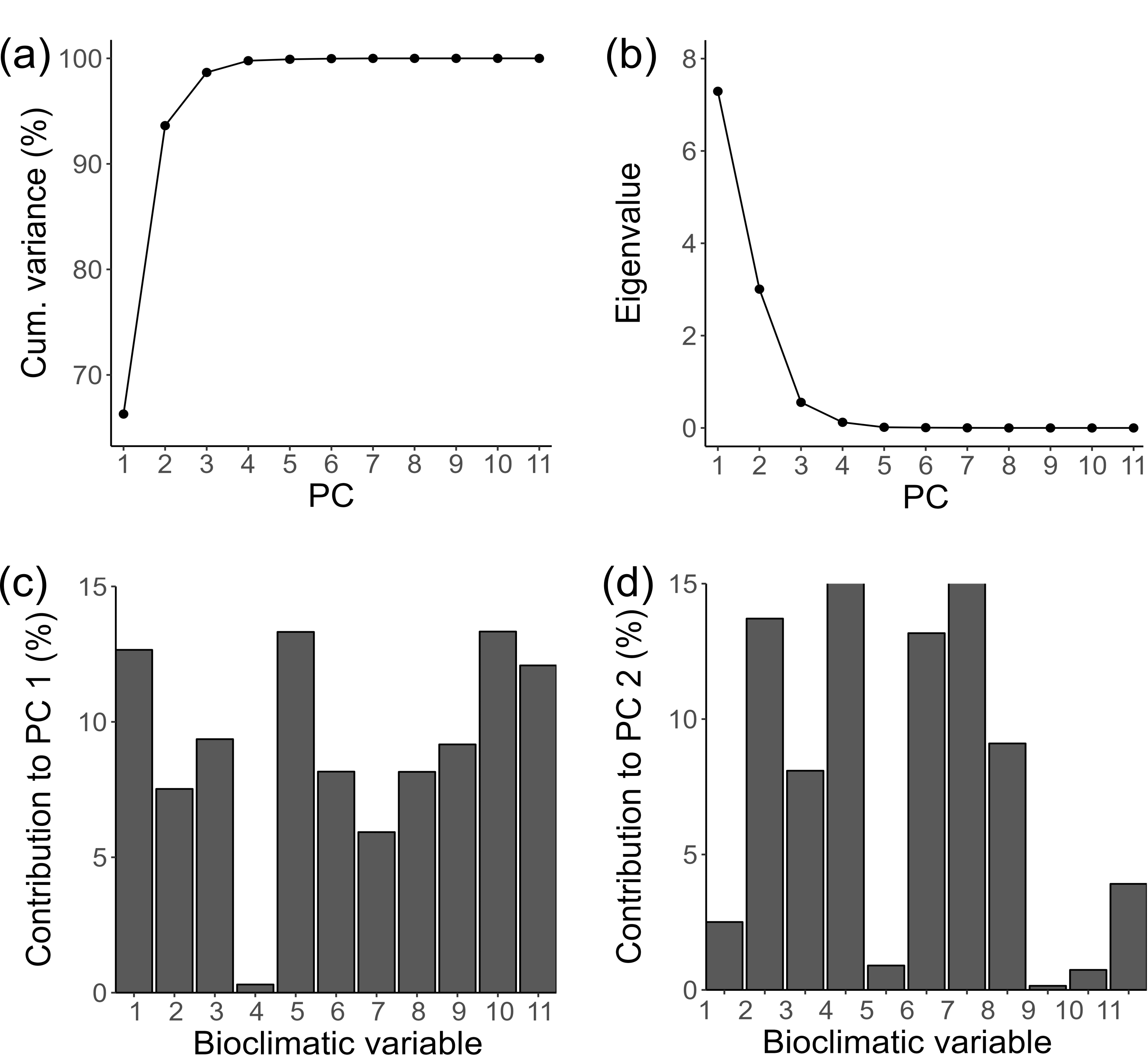
Figure S6.** Statistics related to PCA. (a) Cumulative variance in all temperature-related bioclimatic variables in PC1 to PC11. (b) Eigenvalues of each PC. (c) Percentage contribution of bioclimatic variables to PC1. (d) Percentage contribution of bioclimatic variables to PC2.

**
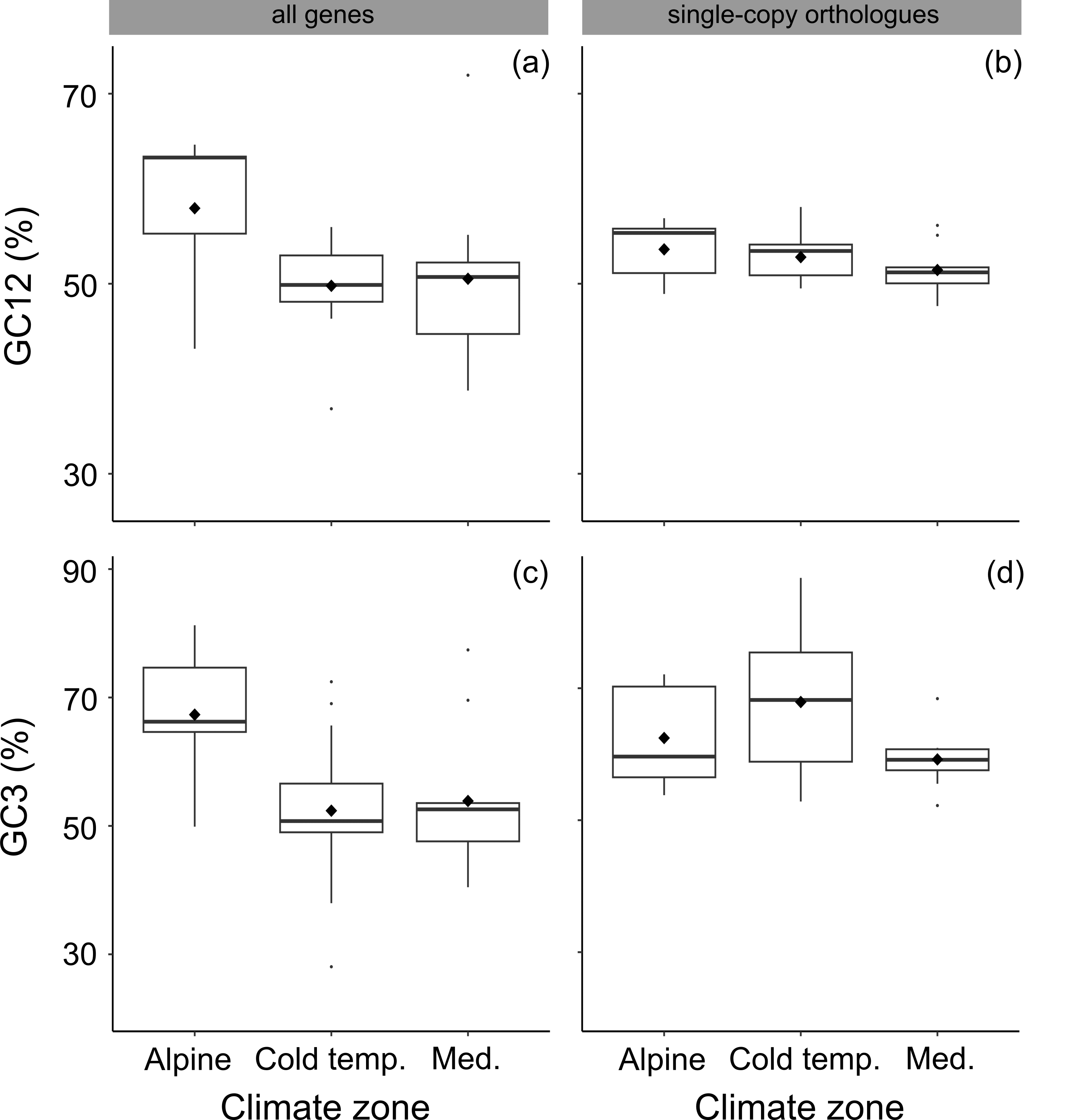
Figure S7.** Differences in GC content in different codon positions in all annotated genes (a, c) and single-copy orthologues (b, d) only.

**
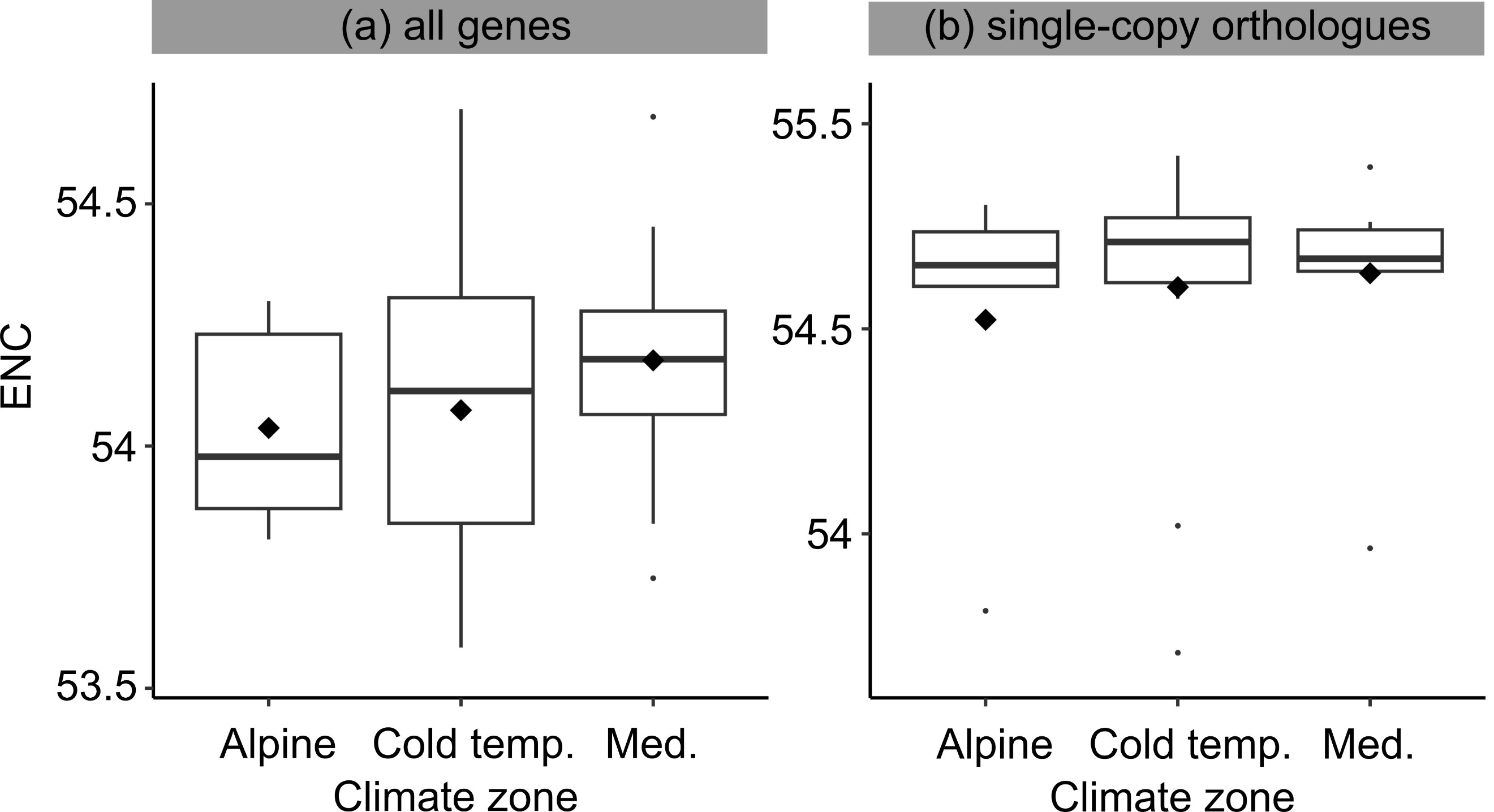
Figure S8.** Comparison of effective number of codons (ENC) among samples collected from the same climate zone in all annotated genes (a) and single-copy orthologues (b). None of the pairwise comparisons were significant.

**
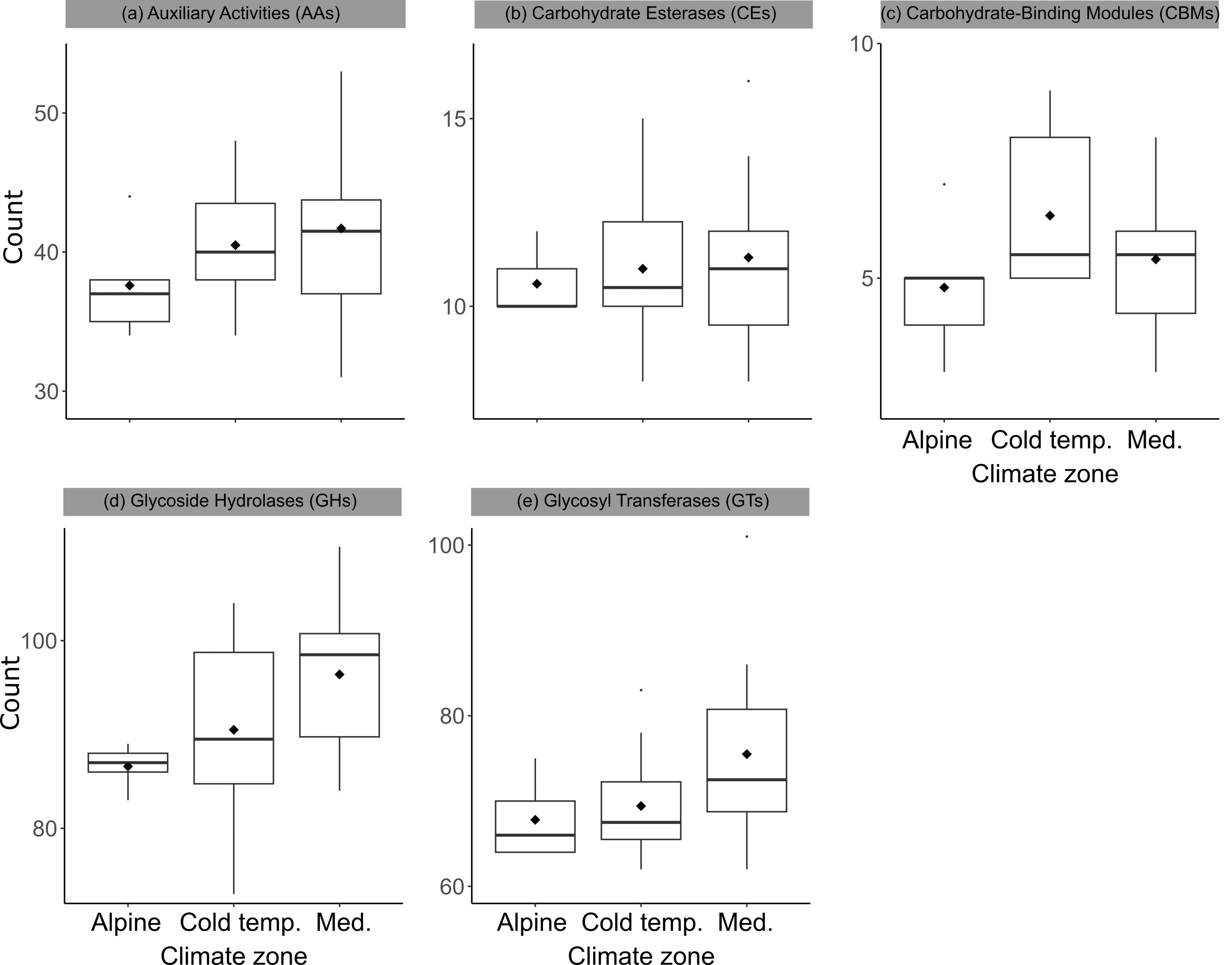
Figure S9.** Distribution of CAZymes in each enzyme class among samples in each climate zone. There is no significant difference between any climate zone pairs.

**
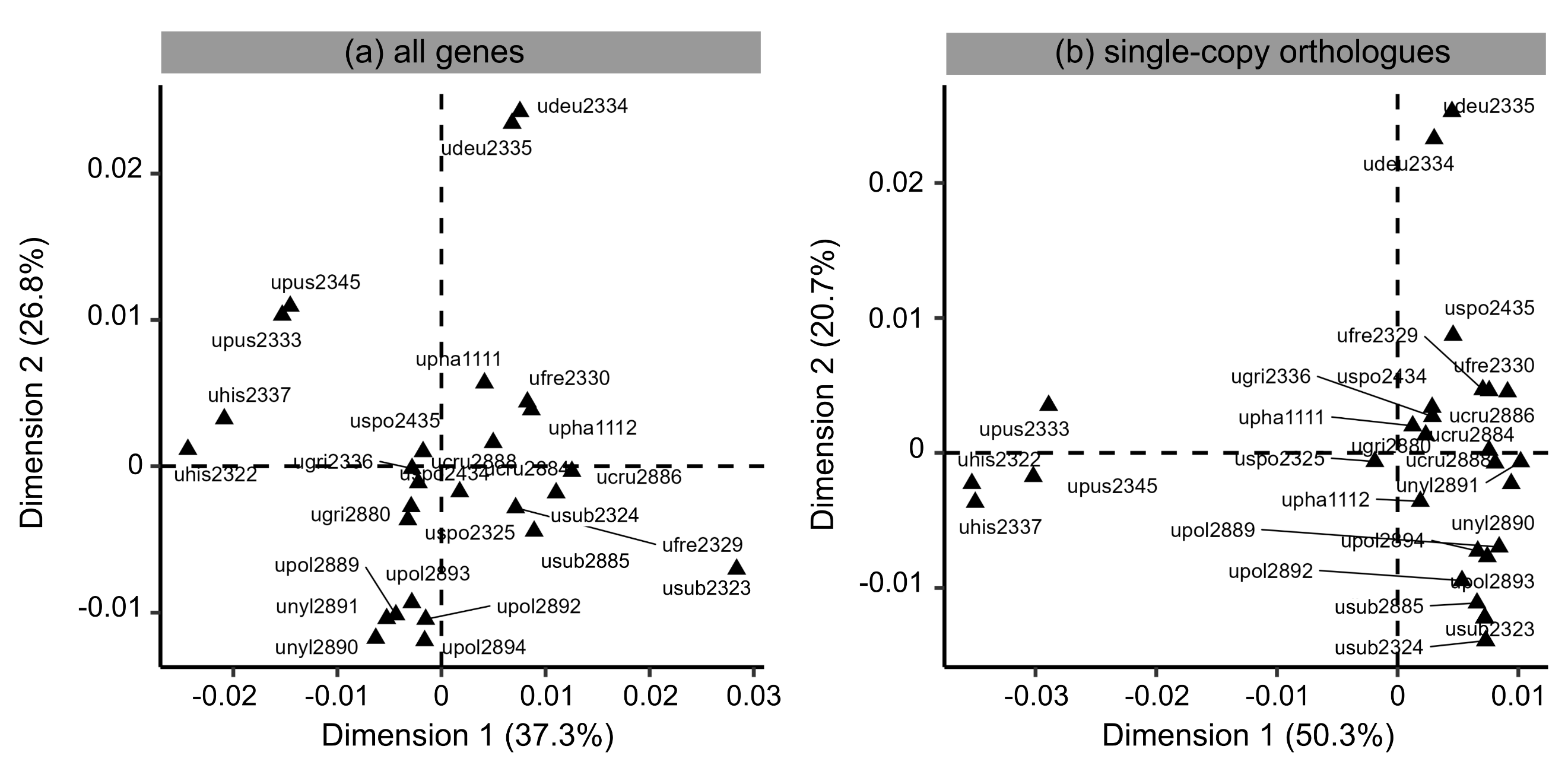
Figure S10.** Correspondence analysis of relative synonymous codon usage (RSCU), displaying similarity in RSCU among the 27 samples in all annotated genes (a) and single-copy orthologues (b).

**
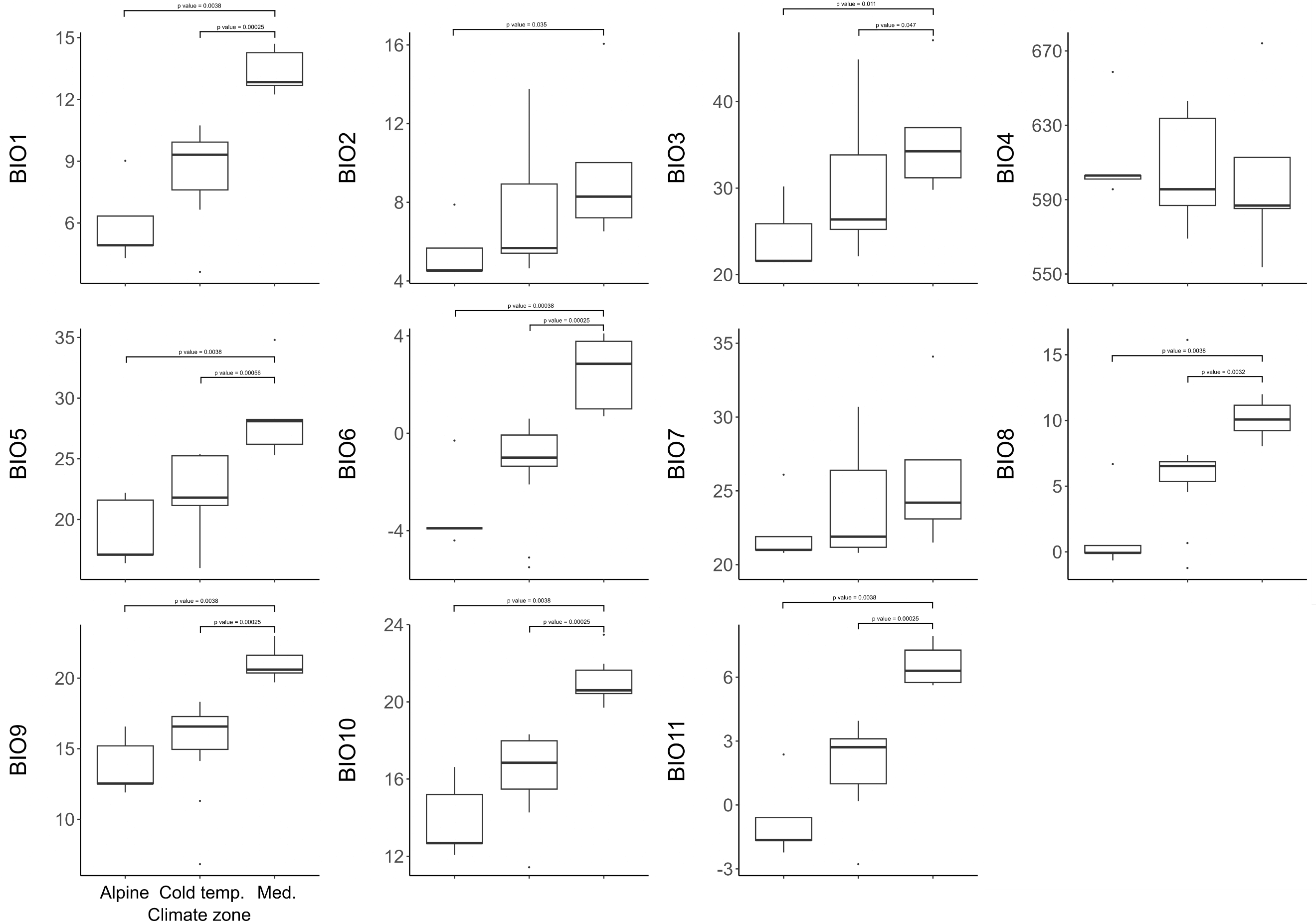
 Figure S11.** Distribution of temperature-related bioclimatic variable values among samples in each climate zone. Only p-values of significant differences are shown.

**
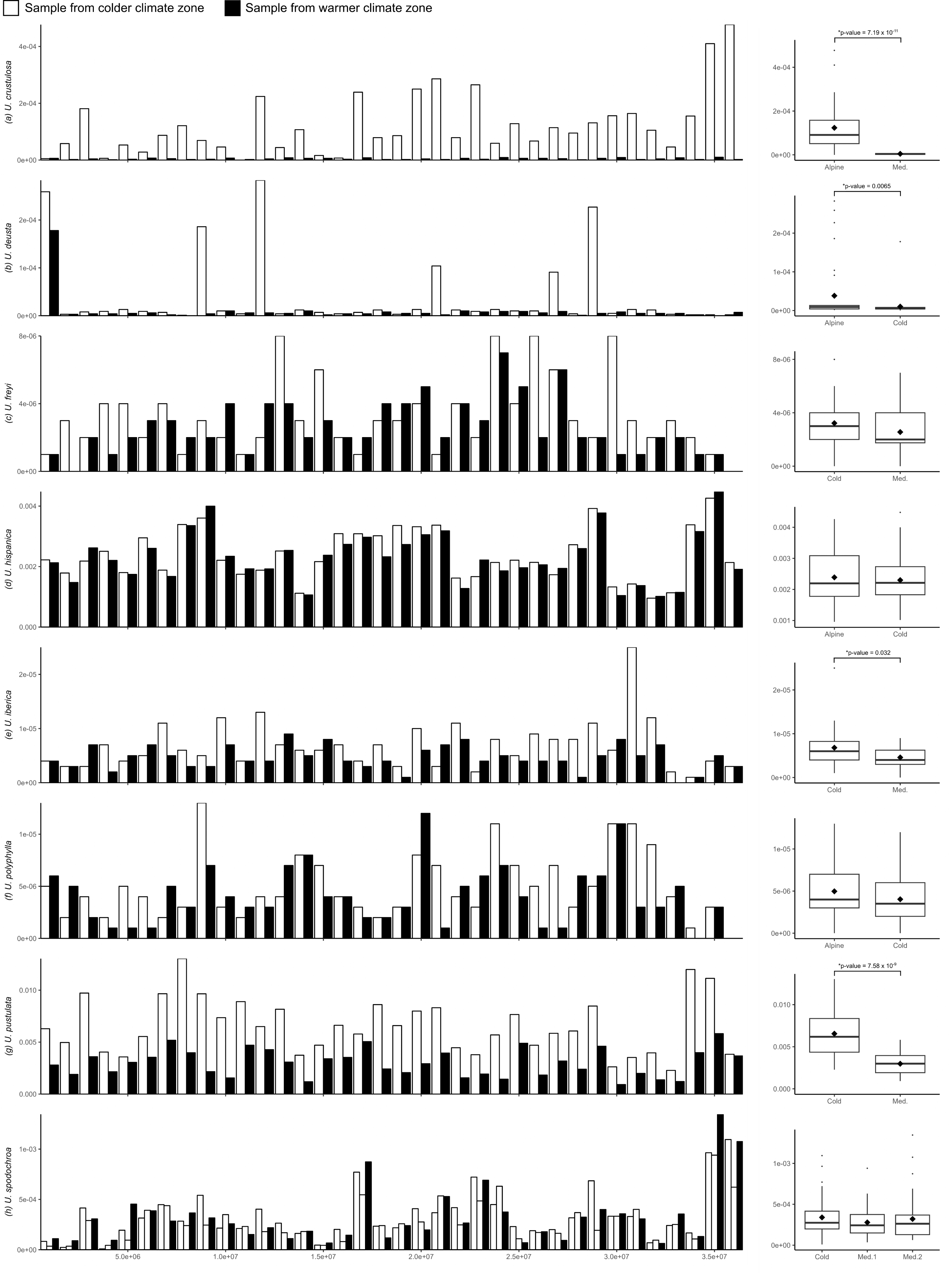
 Figure S12.** Methylation rate between samples collected from different climate zones within the same species. Each horizontal panel contains two figures for each species: the left figure is the methylation rate per window along the genome (non-overlapping 1Mb windows). The right figure is a box plot for comparing the distribution of these methylation rates between the different climate zone. Only significant differences are shown.

**
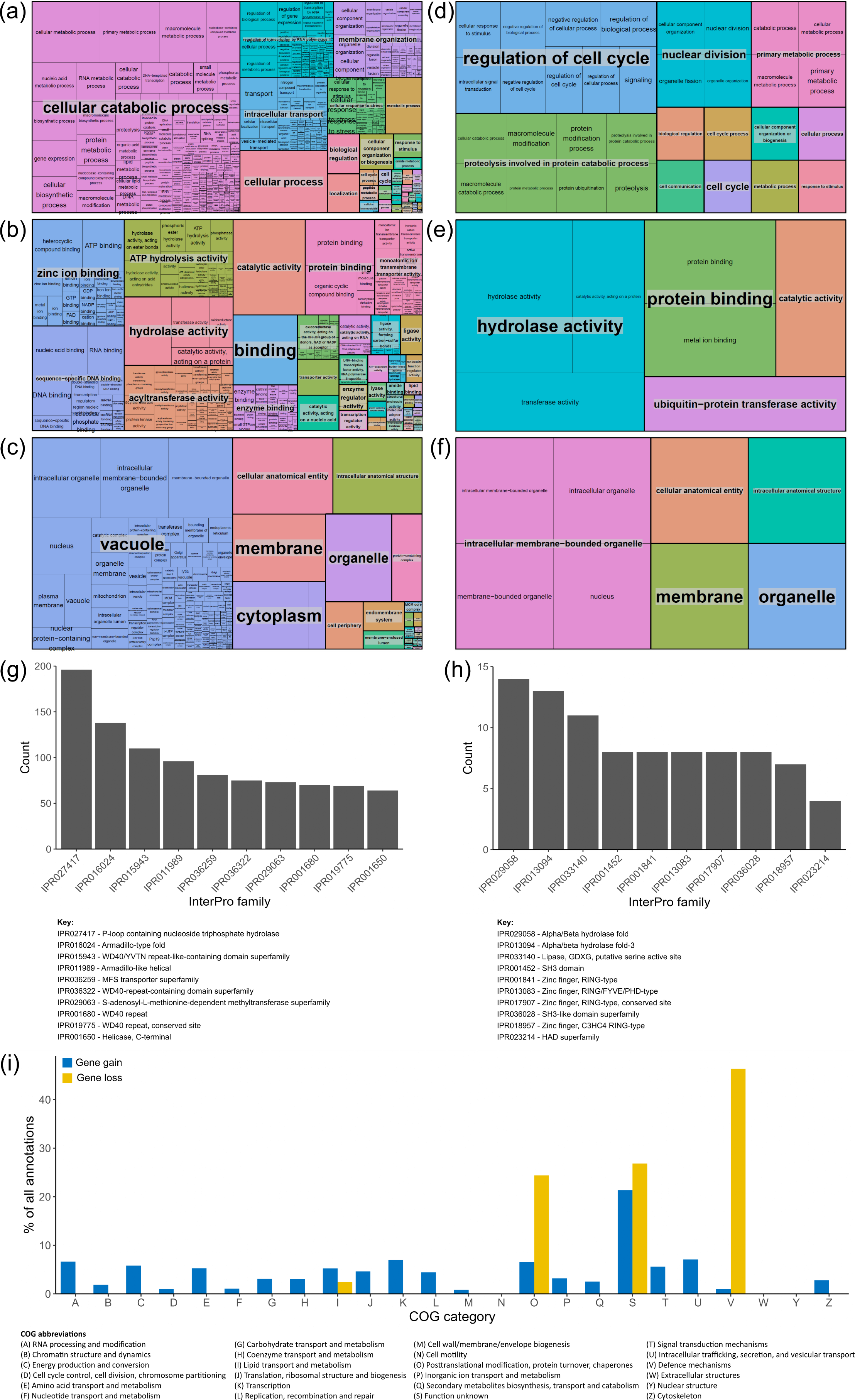
**

**Figure S13.** Annotations of genes in gene families with significant changes in all *U. subpolyphylla* samples compared to *U. polyphylla* samples. (a-f) Summary of GO terms in gene families with significant expansion regarding biological processes (a), molecular functions (b), cellular components (c), gene families with significant contraction regarding biological processes (d), molecular functions (e) and cellular components (f). (g-h) Top 10 annotated InterPro families in gene families with significant expansion (g) and contraction (h). (i) Cluster of orthologues (COGs) of genes.

**
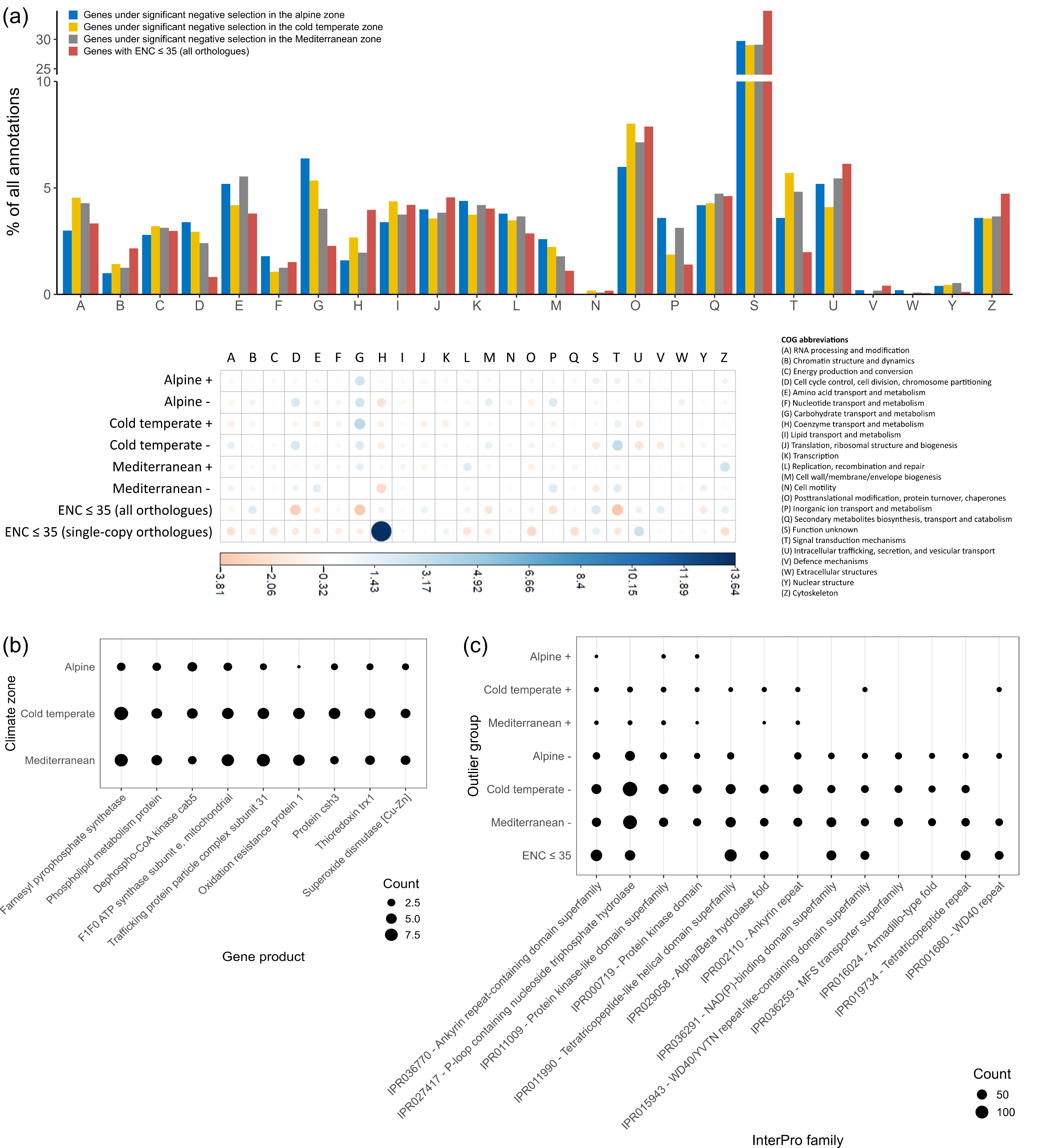
Figure S14.** Comparisons of annotations of outliers among different analysis group. (a) Cluster of orthologues (COGs). Top: Percentage annotated for gene families with significant change, genes under significant negative selection in each climate zone, and genes with strong codon bias (ENC ≤ 35). COGs for genes under significant positive selection are not shown as only very few genes were identified (see Additional File 1: Table S18 for full results). Bottom: Pearson residuals of χ^2^ test of independence in all gene categories. +/- denotes genes under significant positive or negative selection. (b) Gene products encoded from genes with strong codon bias (ENC ≤ 35) in at least ten (out of 27) samples. X-axis is ordered from the most (left, 20 samples) to least (right, ten samples) number of samples in total that fit the criteria. Size of circle denotes the number of samples in each climate zone. (c) Top 20 InterPro protein families annotated for each analysis group, and which are annotated for at least four analysis groups (see Additional File 1: Table S18 for full results). X-axis is ordered from the highest (left, all eight groups) to lowest (right, four groups) number of analysis groups with that InterPro family annotation. Circle sizes denote the number of genes annotated with the respective InterPro protein family.

**
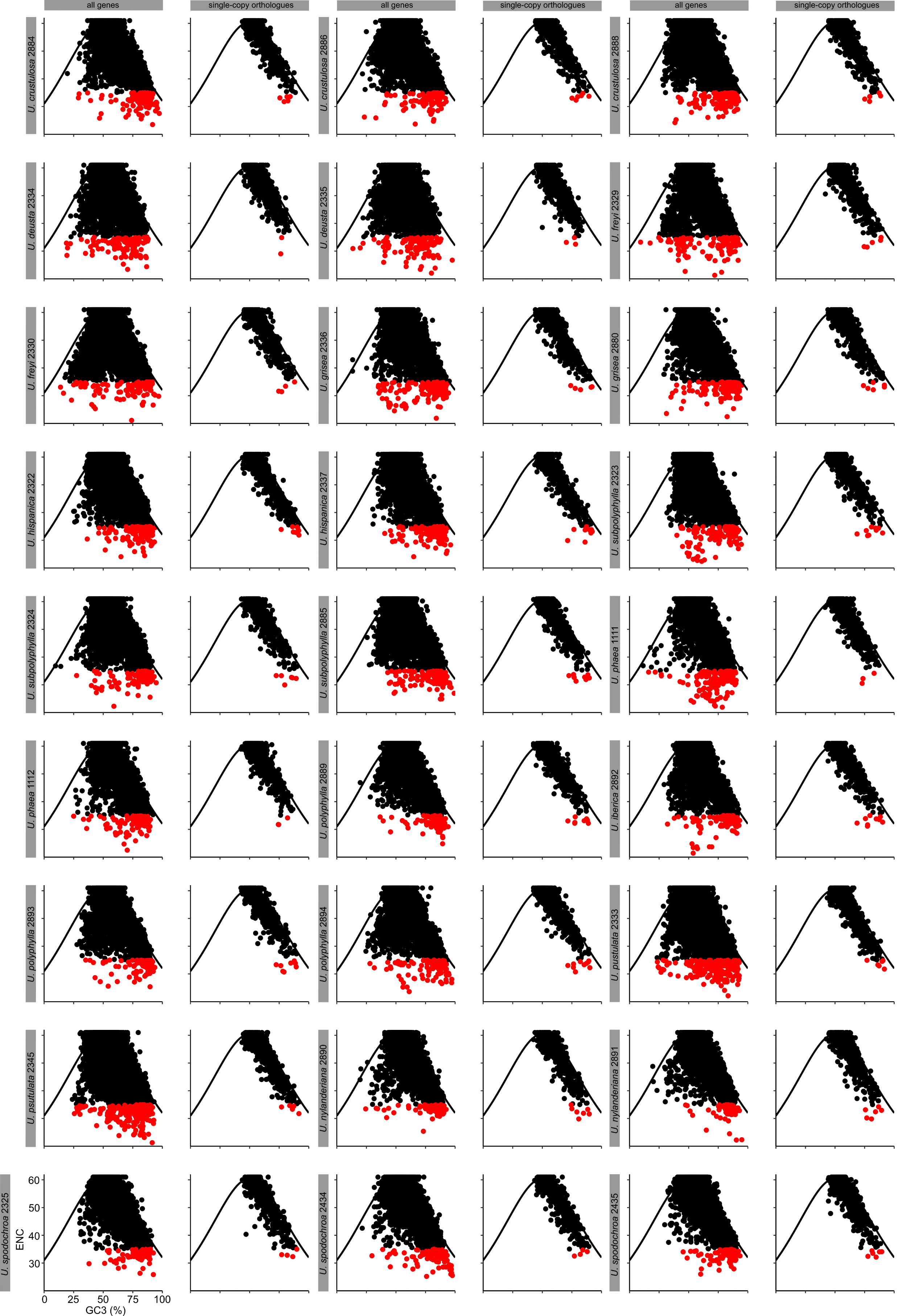
 Figure S15.** Effective number of codons (ENC) of each gene in each species. For each species, left panel shows ENC in all annotated genes; right panel shows ENC in single-copy orthologues only. Red data points denote genes with strong codon bias (ENC ≤ 35). Black curve denotes the expected codon usage if GC compositional constraints alone account for the codon usage bias.
